# The SARS-CoV-2 spike S375F mutation characterizes the Omicron BA.1 variant

**DOI:** 10.1016/j.isci.2022.105720

**Published:** 2022-12-05

**Authors:** Izumi Kimura, Daichi Yamasoba, Hesham Nasser, Jiri Zahradnik, Yusuke Kosugi, Jiaqi Wu, Kayoko Nagata, Keiya Uriu, Yuri L. Tanaka, Jumpei Ito, Ryo Shimizu, Toong Seng Tan, Erika P. Butlertanaka, Hiroyuki Asakura, Kenji Sadamasu, Kazuhisa Yoshimura, Takamasa Ueno, Akifumi Takaori-Kondo, Gideon Schreiber, the Genotype to Phenotype Japan (G2P-Japan) Consortium, Mako Toyoda, Kotaro Shirakawa, Takashi Irie, Akatsuki Saito, So Nakagawa, Terumasa Ikeda, Kei Sato

**Affiliations:** 1 Division of Systems Virology, Department of Microbiology and Immunology, the Institute of Medical Science, the University of Tokyo, Tokyo 1088639, Japan; 2 Faculty of Medicine, Kobe University, Kobe 6500017, Japan; 3 Division of Molecular Virology and Genetics, Joint Research Center for Human Retrovirus Infection, Kumamoto University, Kumamoto 8600811, Japan; 4 Department of Clinical Pathology, Faculty of Medicine, Suez Canal University, Ismailia 41511, Egypt; 5 Department of Biomolecular Sciences, Weizmann Institute of Science, Rehovot 76100, Israel; 6 Graduate School of Medicine, the University of Tokyo, Tokyo 1130033, Japan; 7 Department of Molecular Life Science, Tokai University School of Medicine, Isehara 2591193, Japan; 8 CREST, Japan Science and Technology Agency, Kawaguchi 3220012, Japan; 9 Department of Hematology and Oncology, Graduate School of Medicine, Kyoto University, Kyoto 6068507, Japan; 10 Department of Veterinary Science, Faculty of Agriculture, University of Miyazaki, Miyazaki 8892192, Japan; 11 Division of Infection and Immunity, Joint Research Center for Human Retrovirus Infection, Kumamoto University, Kumamoto 8600811, Japan; 12 Tokyo Metropolitan Institute of Public Health, Tokyo 1690073, Japan; 13 Institute of Biomedical and Health Sciences, Hiroshima University, Hiroshima 7398511, Japan; 14 Center for Animal Disease Control, University of Miyazaki, Miyazaki 8892192, Japan; 15 Graduate School of Medicine and Veterinary Medicine, University of Miyazaki, Miyazaki 8891692, Japan; 16 International Research Center for Infectious Diseases, the Institute of Medical Science, the University of Tokyo, Tokyo 1088639, Japan; 17 International Vaccine Design Center, the Institute of Medical Science, the University of Tokyo, Tokyo 1088639, Japan; 18 Graduate School of Frontier Sciences, the University of Tokyo, Kashiwa 2778561, Japan; 19 Collaboration Unit for Infection, Joint Research Center for Human Retrovirus Infection, Kumamoto University, Kumamoto 8600811, Japan

**Keywords:** Molecular biology, Virology

## Abstract

Recent studies have revealed the unique virological characteristics of Omicron, particularly those of its spike protein, such as less cleavage efficacy in cells, reduced ACE2 binding affinity, and poor fusogenicity. However, it remains unclear which mutation(s) determine these three virological characteristics of Omicron spike. Here, we show that these characteristics of the Omicron spike protein are determined by its receptor-binding domain. Of interest, molecular phylogenetic analysis revealed that acquisition of the spike S375F mutation was closely associated with the explosive spread of Omicron in the human population. We further elucidated that the F375 residue forms an interprotomer pi-pi interaction with the H505 residue of another protomer in the spike trimer, conferring the attenuated cleavage efficiency and fusogenicity of Omicron spike. Our data shed light on the evolutionary events underlying the emergence of Omicron at the molecular level.

## Highlights

• Omicron spike receptor-binding domain determines virological characteristics
• Spike S375F mutation results in the poor spike cleavage and fusogenicity in Omicron
• Acquisition of the spike S375F mutation triggered the explosive spread of Omicron
• F375-H505-mediated π-π interaction in the spike determines the phenotype of Omicron

## Graphical abstract

**Figure:**
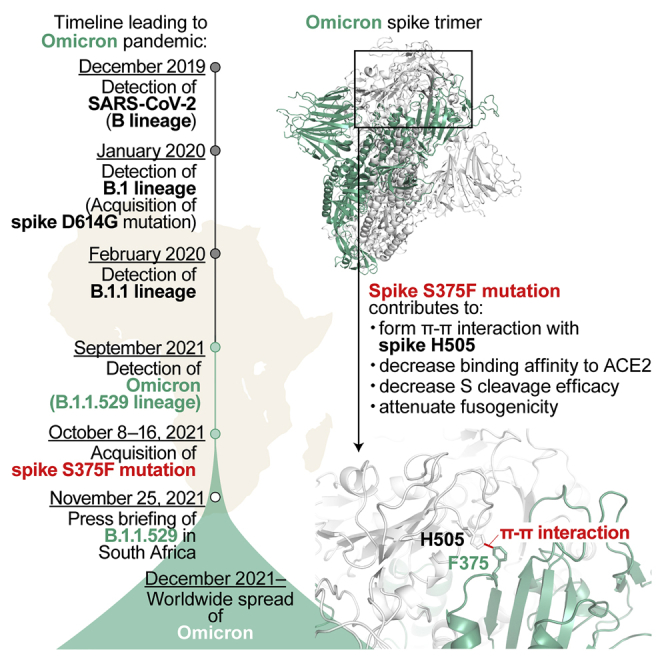

## Introduction

Since the emergence of SARS-CoV-2 at the end of 2019, this virus has become spectacularly diverse. In April 2022, the WHO defined two variants of concern, Delta (B.1.617.2 and AY lineages) and Omicron (originally the B.1.1.529 lineage, then reclassified into BA lineages)^1^; currently, Omicron is the predominant variant spreading worldwide.

Even before detection of the Omicron B.1.1.529 lineage at the end of November 2021 in South Africa,^2^ SARS-CoV-2 had become highly diversified from the original lineage, the B lineage, which was isolated in Wuhan, China, on December 24, 2019 (strain Wuhan-Hu-1, GISAID ID: EPI_ISL_402123).^3^ Regarding the evolutionary scenario leading to the emergence of Omicron, the B.1 lineage, which had acquired the D614G mutation in the spike (S) protein,^4^^,^^5^^,^^6^^,^^7^^,^^8^ was first reported on January 24, 2020 (GISAID ID: EPI_ISL_451345). Thereafter, the B.1.1 lineage was first reported in England on February 16, 2020 (GISAID ID: EPI_ISL_466615). The B.1.1 lineage is the common ancestor of both Alpha (B.1.1.7 lineage), a prior variant of concern by March 2022, and Omicron (B.1.1.529 lineage), and the Alpha variant caused a large surge of infection worldwide beginning in the fall of 2020.^9^ Omicron was first reported in South Africa on September 30, 2021 (GISAID ID: EPI_ISL_7971523).^2^

Soon after the press briefing on Omicron emergence on November 25, 2021,^2^ the virological characteristics of Omicron, currently designated BA.1 (i.e., B.1.1.529.1 lineage, hereafter referred to as Omicron in this study), were intensively investigated. For example, Omicron exhibits profound resistance to the humoral immunity induced by vaccination and natural SARS-CoV-2 infection.^10^^,^^11^^,^^12^^,^^13^^,^^14^^,^^15^^,^^16^^,^^17^^,^^18^^,^^19^^,^^20^^,^^21^ In addition, we demonstrated that the Omicron spike (S) protein is less prone to cleavage by furin, a cellular protease, and exhibits poor fusogenicity.^18^^,^^22^ Moreover, we showed that the binding affinity of the receptor-binding domain (RBD) of Omicron S to human ACE2 is significantly lower than that of ancestral B.1 S.^14^^,^^23^ However, it remains unclear why Omicron has spread so rapidly worldwide. In particular, although the explosive infectious spread of Omicron in the human population can be mainly characterized by the virological properties of Omicron S, the mutation(s) in Omicron S that are responsible for its virological characteristics, such as inefficient S cleavage, lower fusogenicity, reduced ACE2 binding affinity and profound immune resistance, have not been well elucidated.

In this study, we first demonstrate that the representative characteristics of Omicron S, such as immune resistance, poor S cleavage efficiency and poor fusogenicity, are determined by its RBD. Based on molecular phylogenetic analysis, we show that acquisition of the S375F mutation in the Omicron RBD is closely associated with its explosive spread. Moreover, we experimentally demonstrate that the S375F mutation is critical for the virological properties of Omicron S, namely, attenuation of S cleavage efficiency and fusogenicity as well as the decrease in ACE2 binding affinity. Furthermore, we determined how attenuated S cleavage and fusogenicity are conferred by the S375F mutation.

## Results

### The Omicron RBD determines the major virological features of the Omicron variant

**Figure 1:**
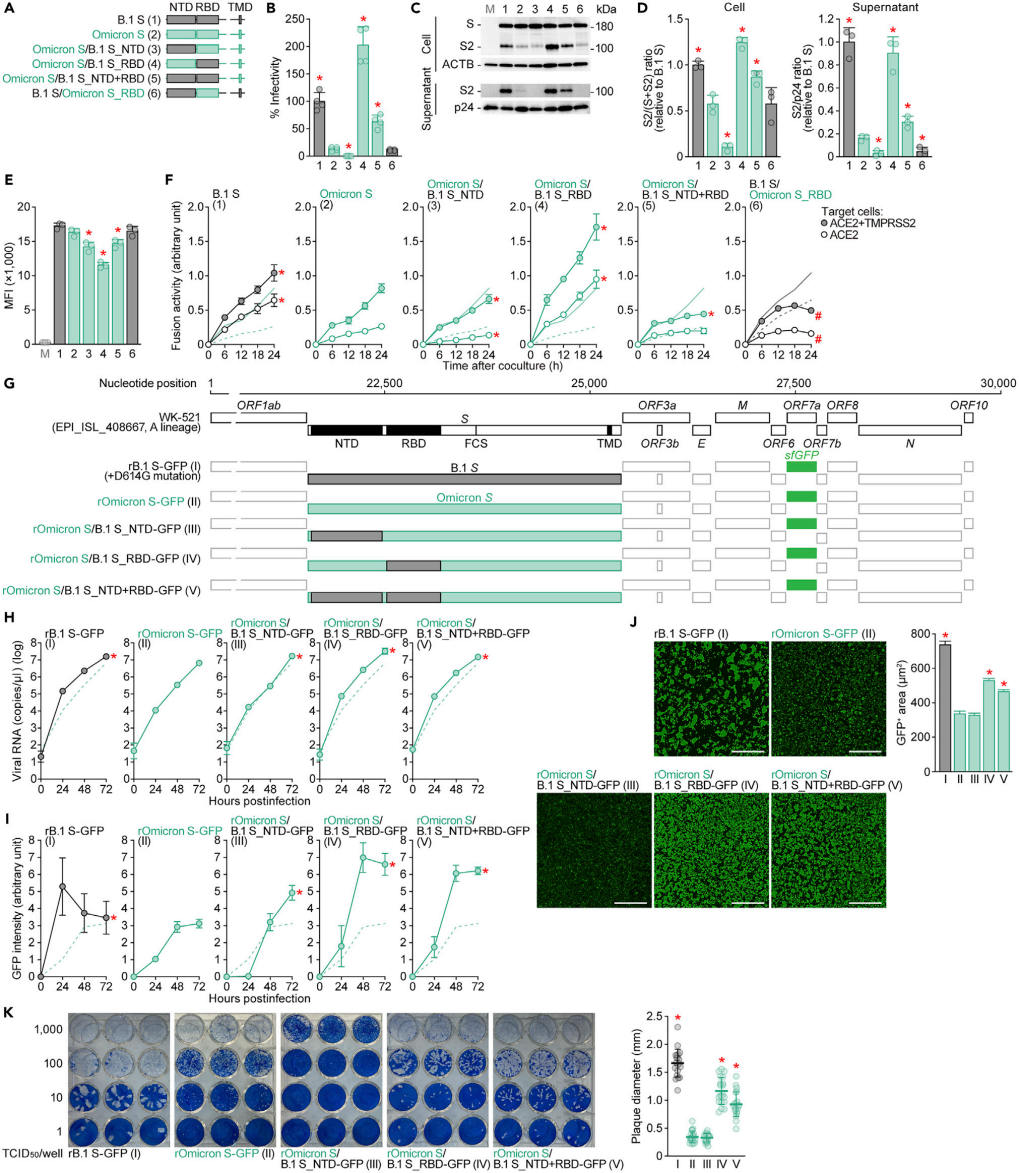
Virological properties conferred by the Omicron RBD (A) Scheme of S chimeras used in this study. The numbers in parentheses are identical to those in Figures 1B–1E and 2. NTD, N-terminal domain; RBD, receptor-binding domain; TMD, transmembrane domain. (B) Pseudovirus assay. HIV-1-based reporter viruses pseudotyped with SARS-CoV-2 S chimeras (summarized in Figure 1A) were prepared. The pseudoviruses were inoculated into HOS-ACE2/TMPRSS2 cells at 1 ng HIV-1 p24 antigen, and the percentages of infectivity compared to that of the virus pseudotyped with B.1 S (spike 1) are shown. (C and D) Western blot. Representative blots of S-expressing cells and supernatants (C) and quantified band intensity (the ratio of S2 to the full-length S plus S2 proteins for “cell”; the ratio of S2 to HIV-1 p24 for “supernatant”) (D) are shown. M, mock (empty vector-transfected). Uncropped blots are shown in Figure S4. (E) Flow cytometry. The summarized results of the surface S expression are shown. MFI, mean fluorescent intensity; M, mock (empty vector-transfected). (F) SARS-CoV-2 S-based fusion assay. The fusion activity was measured as described in the STAR Methods, and fusion activity (arbitrary units) is shown. For the target cells, HEK293 cells expressing ACE2 and TMPRSS2 (filled) and HEK293 cells expressing ACE2 (open) were used. The results for B.1 S or Omicron S are shown in other panels as black and green lines, respectively. The results in HEK293-ACE2/TMPRSS2 cells and HEK293-ACE2 cells are shown as normal or broken lines, respectively. (G) Scheme of the S-chimeric recombinant SARS-CoV-2 used in this study. FCS, furin cleavage site. The backbone is SARS-CoV-2 strain WK-521 (GISAID ID: EPI_ISL_408667, A lineage).^25^ Note that the *ORF7a* gene is swapped with the *sfGFP* gene. The numbers in parentheses are identical to those in Figures 1H–1K. (H–J) SARS-CoV-2 infection. VeroE6/TMPRSS2 cells were infected with a series of chimeric recombinant SARS-CoV-2 (shown in G) at MOI (m.o.i.) 0.01. Viral RNA in the supernatant (H) and GFP intensity (I) were measured using routine techniques. Note that the yaxes of the graphs shown in H are log scales. The result for Omicron (virus II) is shown in other panels as a broken green line. (J) Syncytium formation. Left, GFP-positive area at 48 h.p.i. Scale bar, 500 μm. Right, summarized results. I, n = 6,483 cells; II, n = 5,393 cells; III, n = 8,704 cells; IV, n = 13,188 cells; and V, n = 12,749 cells. Representative images are shown in Figure S1. (K) Plaque assay. Left, representative figures.Right, summary of the plaque diameters (20 plaques per virus). Data are expressed as the mean with SD (B, D-F, and H–K) or the median with 95% confidence interval (CI) (J). Assays were performed in quadruplicate (B, H, and I) or triplicate (D–F). Each dot indicates the result of an individual replicate (B, D and E) or an individual plaque (K). Statistically significant differences (∗p <0.05) versus Omicron S (pseudovirus 2 for B, D and E, virus II for J and K) were determined by two-sided Student’s *t* test (B and E), two-sided paired *t* test (D), or two-sided Mann–Whitney U test (J and K). In F, H and I, statistically significant differences versus Omicron (spike 2 or virus II) [∗familywise error rates (FWERs)<0.05] (except for the rightmost panel in **F**) or B.1 (spike 1 or virus I) [#familywise error rates (FWERs)<0.05] (rightmost panel in **F**) through timepoints were determined by multiple regression. FWERs were calculated using the Holm method. See also Figures S1 and S4.

To determine the mutation(s) responsible for the virological features of Omicron, we prepared a series of expression plasmids for Omicron S-based chimeric mutants with swapping of the N-terminal domain (NTD) and/or RBD of B.1 (D614G-bearing strain) S (Figure 1A). Experiments showed that pseudoviruses with B.1 RBD-bearing Omicron S [Omicron S/B.1 S_RBD (spike 4 in Figure 1A) and Omicron S/B.1 S_NTD+RBD (spike 5)] exhibited increased infectivity compared to pseudovirus with Omicron S (spike 2) in HOS-ACE2/TMPRSS2 cells (Figure 1B) and A549-ACE2 cells (Figure S1A). Western blot analysis (Figure 1C) showed that the S cleavage efficacy in cells (Figure 1D, left) correlated with the level in virion-incorporated S2 protein (Figure 1D, right) and pseudovirus infectivity (Figure 1B). In particular, the cleavage efficacy of Omicron S was lower than that of B.1 S, which is consistent with our recent studies (spikes 1 and 2 in Figures 1C and 1D).^18^^,^^22^^,^^23^ On the other hand, chimeric Omicron S proteins bearing the B.1 RBD (spikes 4 and 5) displayed increased cleavage efficacy (Figures 1C and 1D). Although the surface expression levels of a series of Omicron S chimeras bearing the B.1 domains (spikes 3–5) were lower than those of Omicron S chimeras (Figure 1E), a cell-based fusion assay^18^^,^^22^^,^^23^^,^^24^ revealed that the fusogenicity of B.1 RBD-bearing Omicron S was significantly higher than parental Omicron S (Figure 1F). To verify the importance of the RBD for the phenotype of Omicron S, we performed reversal experiments based on B.1 S [B.1 S/Omicron S_RBD (spike 6) in Figure 1A]. Corresponding to the results for Omicron S, the pseudovirus infectivity (Figure 1B), S cleavage efficacy (Figures 1C and 1D), and fusogenicity (Figure 1F) of Omicron RBD-harboring S [B.1 S/Omicron S_RBD (spike 6)] were attenuated compared to those of parental B.1 S. These results suggest that the RBD of Omicron S mainly determines the attenuated cleavage efficacy and decreased fusogenicity of Omicron S.

To further investigate the impact of the Omicron S RBD on multicycle viral replication, we generated a series of recombinant chimeric SARS-CoV-2 strains by reverse genetics (Figure 1G).^25^ As shown in Figure 1H, the growth of rOmicron S-GFP (virus II) and rOmicron S/B.1 S_NTD (virus III) was lower than that of rB.1 S-GFP (virus I). In sharp contrast, recombinant viruses bearing the B.1 RBD [rOmicron S/B.1_RBD-GFP (virus IV) and rOmicron S/B.1 S_NTD+RBD-GFP (virus V)] replicated more efficiently than rOmicron S-GFP (virus II) in VeroE6/TMPRSS2 cells (Figure 1H). In addition, to monitor the spread of these recombinant viruses, we measured GFP intensity in infected cell cultures. We found that the GFP intensity of cells infected with recombinant viruses bearing the B.1 RBD was significantly higher than that of cells infected with rOmicron S-GFP (virus II) (Figures 1I and S1B). These data suggest that the RBD of Omicron S attenuates viral growth capacity in cell cultures. We then measured the number of GFP-positive cells to evaluate the fusogenicity of the chimeric viruses. As shown in Figure 1J, the GFP-positive area of cells infected with the recombinant viruses at 48 h post infection (h.p.i.) was significantly larger for viruses bearing the B.1 RBD [rOmicron S/B.1_RBD-GFP (virus IV) and rOmicron S/B.1 S_NTD+RBD-GFP (virus V)] than for rOmicron-GFP (virus II). Consistent with the results in cells transfected with S expression plasmids (Figure 1F), these findings suggest that the Omicron RBD attenuates viral fusogenicity. Moreover, the plaques formed by infection with rOmicron S/B.1 S_RBD-GFP (virus IV) and rOmicron S/B.1 S_NTD+RBD-GFP (virus V) were significantly larger than those formed by rOmicron S-GFP virus (virus II), though the plaques formed by rOmicron S-GFP (virus II) and rOmicron S/B.1 S_NTD-GFP (virus III) were comparable (Figure 1K). Altogether, these results suggest that the Omicron RBD determines the virological features of this viral lineage, such as the observed attenuation of S1/S2 cleavage efficacy and fusogenicity.

### The Omicron RBD mainly determines the immune resistance of Omicron

**Figure 2:**
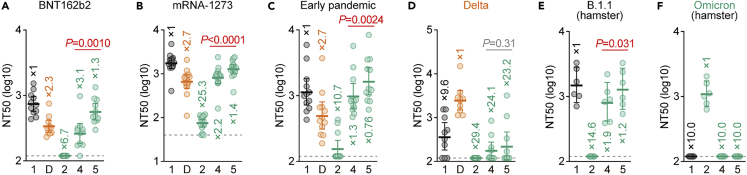
Immune resistance conferred by the Omicron RBD Neutralization assays were performed with pseudoviruses harboring a series of S protein sequences (summarized in Figure 1A). The numbers are identical to those in Figure 1A. D, Delta variant. Vaccinated sera [BNT162b2 (A, 11 donors); or mRNA-1273 (B, 16 donors)], convalescent sera of individuals infected with an early pandemic virus (before May 2020) (C, 12 donors), or Delta (D, 10 donors) and convalescent sera of hamsters infected with B.1.1 (E, 6 hamsters)^22^ or Omicron (F, 6 hamsters)^22^ were used. The list of sera used in this experiment is shown in Table S1. Each serum sample was analyzed in triplicate to determine the 50% neutralization titer (NT50). Each dot represents one NT50 value, and the geometric mean and 95% CI are shown. The numbers indicate the fold changes of resistance versus each antigenic variant. Horizontal gray lines indicate the detection limit of each assay (120 for A and C–F; 40 for B). Statistically significant differences between spikes 4 and 5 were determined by a two-sided Wilcoxon signed-rank test. See also Table S1.

We next assessed the domains of Omicron S that are associated with the profound immune resistance of Omicron.^10^^,^^11^^,^^12^^,^^13^^,^^14^^,^^15^^,^^16^^,^^17^^,^^18^^,^^19^^,^^20^^,^^21^ Because swapping of Omicron S with the B.1 S NTD (Omicron S/B.1 S_NTD, spike 3) severely decreased pseudovirus infectivity (Figure 1B), we performed neutralization assays using pseudoviruses with Omicron RBD-bearing B.1 S [Omicron S/B.1 S_RBD (spike 4)] and Omicron S/B.1 S_NTD+RBD (spike 5) as well as the S proteins of Omicron (spike 2), Delta and B.1 (spike 1) (the list of sera used is shown in Table S1). Consistent with recent studies,^10^^,^^11^^,^^12^^,^^13^^,^^14^^,^^15^^,^^16^^,^^17^^,^^18^^,^^19^^,^^20^^,^^21^ Omicron S (spike 2) was highly resistant to the vaccine sera [BNT162b2 (Figure 2A) and mRNA-1273 (Figure 2B)] as well as convalescent sera from individuals infected with early-pandemic virus (collected before May 2020) (Figure 2C) or the Delta variant (Figure 2D). Pseudoviruses with the Omicron S/B.1 S_RBD (spike 4) and Omicron S/B.1 S_NTD+RBD (spike 5) were significantly more sensitive to vaccine sera (Figures 2A and 2B) and convalescent sera obtained from early-pandemic virus-infected patients than was Omicron S (spike 2) (Figure 2C). These results suggest that the RBD of Omicron S is closely associated with its pronounced resistance to the antiviral humoral immunity elicited by vaccination or previous SARS-CoV-2 infection. Moreover, we used convalescent sera from hamsters infected with B.1.1 (note that the S gene sequences of B.1 and B.1.1 are identical) and Omicron, as collected in our previous study,^22^ for the assay. As shown in Figure 2E, Omicron S (spike 2) was completely resistant to the B.1.1 convalescent sera, whereas it was sensitive to the Omicron convalescent sera. Notably, chimeric Omicron S bearing the B.1 RBD [Omicron S/B.1 S_RBD (spike 4) and Omicron S/B.1 S_NTD+RBD (spike 5)] exhibited the opposite results: these chimeric pseudoviruses were sensitive to the B.1.1 convalescent sera (Figure 2E) but completely resistant to the Omicron convalescent sera (Figure 2F). These results further suggest that the Omicron RBD determines its immune resistance and is an immunodominant epitope for inducing humoral immunity. However, we found that Omicron S/B.1 S_NTD+RBD (spike 5) is significantly more sensitive to antisera than is Omicron S/B.1 S_RBD (spike 4) (Figures 2A–2C and 2E). These findings suggest that mutations in the NTD of Omicron S are also partly associated with the immune resistance of Omicron S.

### The S S375F mutation increases binding affinity to human ACE2

**Figure 3:**
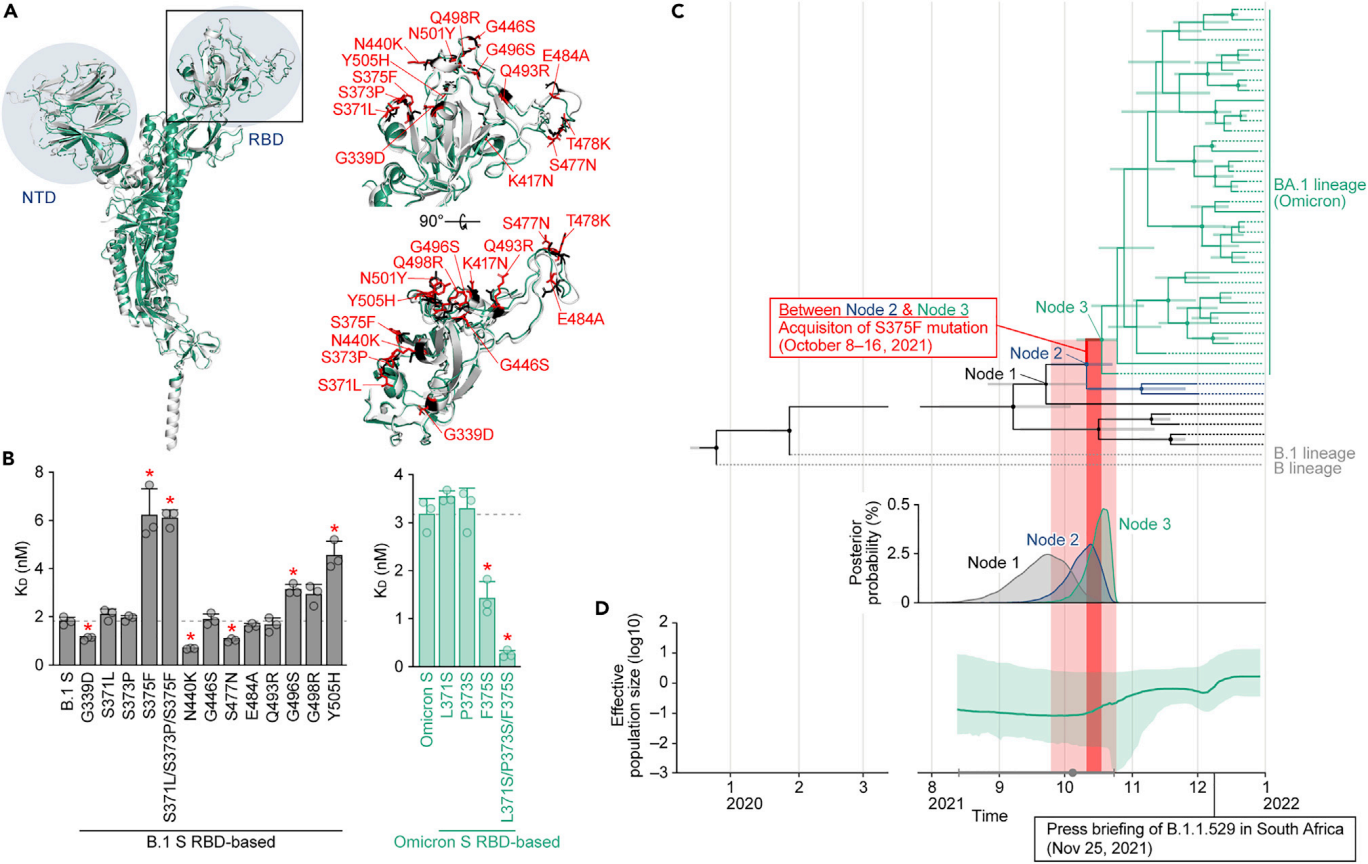
Mutations in the Omicron RBD and the evolution of Omicron (A) Structural insights into the mutations in the Omicron RBD. Left, overlaid cryo-EM structures of SARS-CoV-2 B.1 S (PDB: 7KRQ)^28^ (white) and Omicron S (PDB: 7T9J)^29^ (green) are shown. The NTD and RBD are indicated in blue. The region in the RBD indicated by a square is enlarged in the top right panel. Right, mutated residues in the RBD. The residues in B.1 S and Omicron S are shown in black and red, and the mutations in Omicron S are indicated. (B) ACE2 binding affinity of a series of SARS-CoV-2 S RBD (residues 336–528) mutants tested by yeast surface display. The K_D_ values of the binding of the SARS-CoV-2 S RBD expressed on yeast to soluble ACE2 are shown. (C and D) Evolution of Omicron. (C) Top, a time tree of 44 Omicron variants and two outgroups (B and B.1 lineages). The same tree annotated with the GISAID ID, PANGO lineage and sampling date at each terminal node is shown in Figure S2. Green, Omicron variants containing the S371L, S373P and S375F mutations; blue, Omicron variants containing the S371L and S373P mutations; black, Omicron variants without the S371L/S373P/S375F mutations; and gray, the two outgroups (B and B.1 lineages). The bars on each internal node indicate the 95% highest posterior density (HPD) interval of the estimated time. The size of the circle on each internal node is proportional to the value of posterior probability. Note that “Node 1” corresponds to the time to before the emergence of the S371L and S373P mutations; “Node 2” corresponds to the time after the acquisition of the S371L and S373P mutations and before the emergence of the S375F mutations; and “Node 3” corresponds to the fixation time of the S371L/S373P/S375F mutations in the Omicron variants. The estimated time of each node is as follows: Node 1, September 23, 2021 (95% HPD August 26, 2021 to October 10, 2021); Node 2, October 8, 2021 (95% HPD September 24, 2021 to October 21, 2021); and Node 3, October 16, 2021 (95% HPD October 5, 2021 to October 23, 2021). Bottom, distribution of the posterior probability of the time to the tMRCA of Node 1 (black), Node 2 (blue), and Node 3 (green). (D) Bayesian skyline plot showing the history of the effective population size of 44 Omicron variants. The 95% HPD is shaded in green. The dot (in gray) indicates the estimated tMRCA of the 44 variants (October 5, 2021), and the error bar (in gray) indicates the lower (August 13, 2021) and upper (October 23, 2021) boundaries of the 95% HPD tMRCA. In B, the data are expressed as the mean with SD. The assay was performed in triplicate, and each dot indicates the result of an individual replicate. The horizontal broken lines indicate the value of B.1 S (left) and Omicron S (right), respectively. Statistically significant differences (∗p <0.05) versus B.1 S (left) or Omicron S (right) were determined by two-sided Student’s t tests, and FWERs were calculated using the Holm method. In C and D, the estimated time of S375F emergence [i.e., between “Node 2” and “Node 3” (October 8–16, 2021) in C] is shaded in dark red. The lower and upper boundaries of the 95% HPD tMRCA of “Node 2" and “Node 3”, respectively (i.e., September 24 to October 23, 2021) is shaded in light red. See also Figure S2.

Twelve substitutions are uniquely present in the RBD (residues 319–541) of Omicron S; another 3 substitutions (K417N, T478K and N501Y) are common among the other variants (Figure 3A).^18^ To determine the residue(s) responsible for the virological phenotype of Omicron, particularly the reduced binding affinity of the Omicron S RBD to human ACE2,^14^^,^^23^ we prepared a series of B.1 S RBD point mutants that bear the respective mutations of Omicron and conducted screening experiments based on a yeast surface display assay.^14^^,^^23^^,^^24^^,^^26^^,^^27^ As shown in Figure 3B (left panel), compared to the RBD of parental (i.e., B lineage-based) S, the K_D_ values of the G339D, N440K and S477N mutants were significantly decreased, whereas those of the S375F, S371L/S373P/S375F, G496S and Y505H mutants were significantly increased. Altogether, these data suggest that the S375F, G496S and Y505H substitutions are closely associated with the reduced binding affinity of the Omicron S RBD to human ACE2.

### Omicron emergence is closely associated with acquisition of the S S375F mutation

The S375F, G496S and Y505H mutations in the S protein are almost exclusive to Omicron variants (Table S2). To infer the evolutionary sequence of the emergence of these mutations in the Omicron lineage, we generated a time tree of 44 Omicron genomes detected in 2021 (for more detail, see STAR Methods) (Figures 3C and S2). The G496S and Y505H mutations were detected in all sequences used in this analysis, suggesting that these two mutations were present in the common ancestor of all Omicron variants reported thus far. In contrast, the S371L, S373P and S375F mutations are not present in the older Omicron sequences (shown in black in Figures 3C and S2). Although the emergence times of S371L and S373P cannot be estimated independently, our analysis assumed that the S371L and S373P mutations were first acquired between Node 1 [95% highest posterior density (HPD): August 26, 2021 to October 10, 2021] and Node 2 (95% HPD: September 24, 2021 to October 21, 2021) in Figure 3C, as based on the estimated time to the most recent common ancestor (tMRCA). The S375F mutation emerged thereafter, between Node 2 and Node 3 (95% HPD: October 5, 2021 to October 23, 2021) (Figure 3C). Of interest, the Bayesian skyline plot of the 44 Omicron genomes suggested that the effective population size of Omicron increased around the time of S375F substitution acquisition (Figure 3D). These data suggest that the emergence of the S375F mutation might have been a crucial event triggering the massive spread of Omicron variants in the human population.

To verify the possibility that the S375F mutation is crucial for the phenotype of Omicron, we performed yeast binding assays using the RBD of Omicron S. As depicted in Figure 3B (right panel), the F375S and L371S/P373S/F375S mutations in the RBD of Omicron S significantly increased binding affinity to human ACE2. Overall, these observations suggest that the three substitutions at positions 371, 373 and 375, particularly the S375F substitution, determine the reduced binding affinity of the Omicron S RBD to human ACE2.

### The S S375F mutation determines the S cleavage efficacy, fusogenicity, and ACE2 binding affinity of the Omicron variant

**Figure 4:**
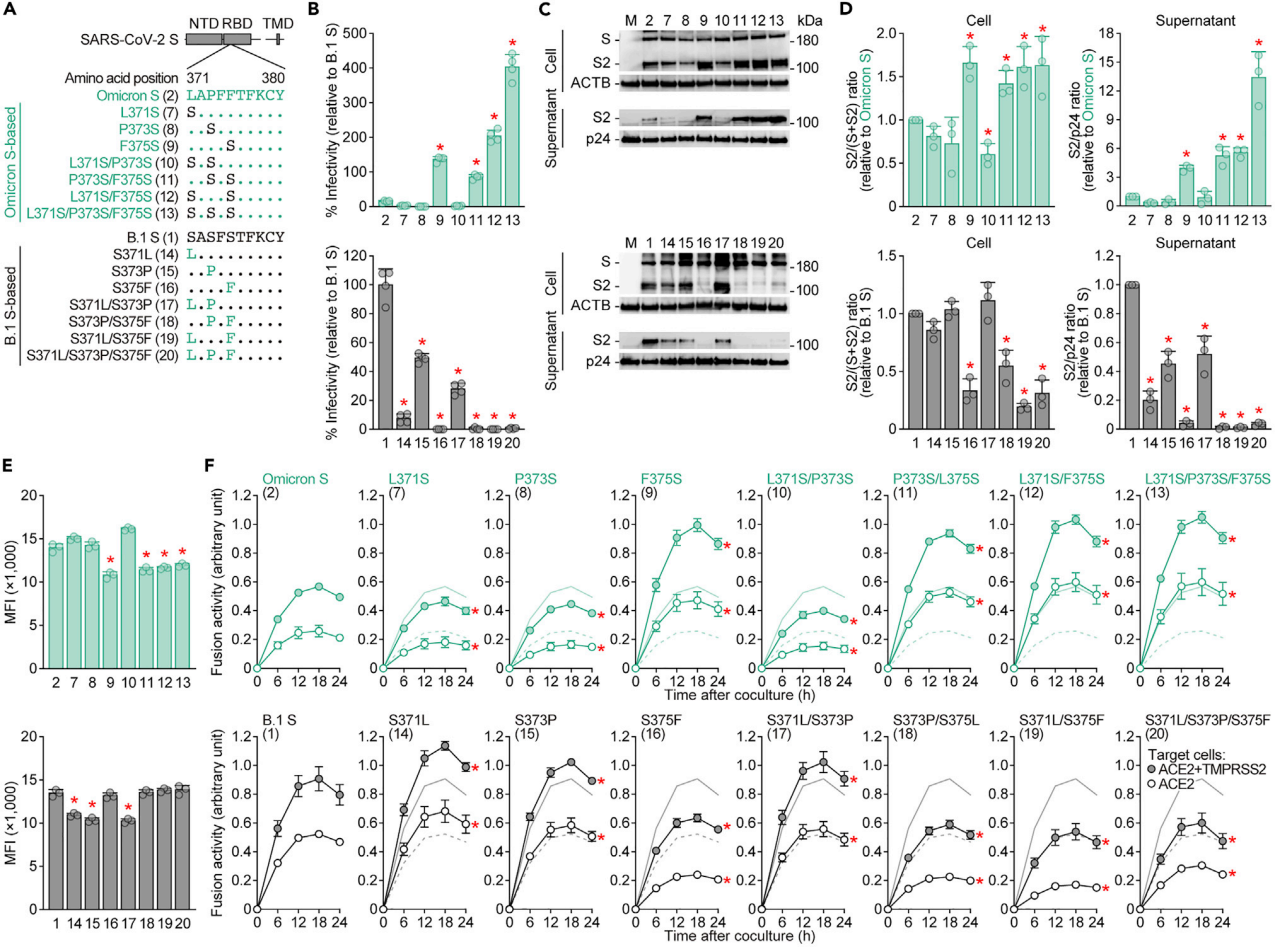
Virological features conferred by the S S375F mutation (A) Scheme of the S mutants used in this study. The numbers in parentheses are identical to those in Figures 4B–4F and S3. (B) Pseudovirus assay. HIV-1-based reporter viruses pseudotyped with SARS-CoV-2 S mutants (summarized in A) were prepared. The pseudoviruses were inoculated into HOS-ACE2/TMPRSS2 cells at 1 ng HIV-1 p24 antigen, and the percent infectivity compared to that of the virus pseudotyped with Omicron S (spike 2, top) or B.1 S (spike 1, bottom) are shown. (C and D) Western blot. Representative blots of S-expressing cells and supernatants (C) and quantified band intensity (the ratio of S2 to the full-length S plus S2 proteins for “cell”; the ratio of S2 to HIV-1 p24 for “supernatant”) (D) are shown. M, mock (empty vector-transfected). Uncropped blots are shown in Figure S4. (E) Flow cytometry. The summarized results of the surface S expression are shown. (F) SARS-CoV-2 S-based fusion assay. The fusion activity was measured as described in STAR Methods, and fusion activity (arbitrary units) is shown. For the target cells, HEK293 cells expressing ACE2 and TMPRSS2 (filled) and HEK293 cells expressing ACE2 (open) were used. The results for Omicron S (top) or B.1 S (bottom) are shown in other panels as green and black lines, respectively. The results in HEK293-ACE2/TMPRSS2 cells and HEK293-ACE2 cells are shown as normal and broken lines, respectively. Data are expressed as the mean with SD. Assays were performed in quadruplicate (B) or triplicate (D–F). In B, D and E, each dot indicates the result of an individual replicate. Statistically significant differences (∗p <0.05) versus the respective parental S [Omicron S (pseudovirus 2, top panels) or B.1 S (spike 1, bottom panels)] were determined by two-sided Student’s *t* test (B and E) or two-sided paired *t* test (D). In F, statistically significant differences (∗FWERs<0.05) versus the respective parental S [Omicron S (spike 2, top panels) or B.1 S (spike 1, bottom panels)] through timepoints were determined by multiple regression. FWERs were calculated using the Holm method. See also Figures S3 and S4.

To investigate the impact of the S375F mutation, we prepared pseudoviruses with a series of Omicron S-based mutations (Figure 4A). In the yeast surface display assay (Figure 3B), the assay based on Omicron S showed that pseudovirus infectivity was clearly increased by the Omicron S F375S mutation (spikes 9 and 11–13 in Figure 4A) (Figure 4B, top). Western blot analysis showed that the S1/S2 cleavage efficacy and level of S2 in virions were rescued by the F375S mutation (Figures 4C and 4D, top). Similar to the results illustrated in Figures 1C and 1D, the mutated S proteins that were efficiently cleaved in cells (e.g., spikes 9 and 11–13) were also efficiently incorporated into the viral particles released (Figures 4C and 4D). These results indicate that the level of virion-incorporated S2 is modulated by the S cleavage efficacy in producer cells and that pseudovirus infectivity can be an indicator of the level of S protein cleavage in producer cells. Although the surface S expression level was decreased by the F375S mutation (Figure 4E, top), a cell-based fusion assay demonstrated that the mutation significantly increased the efficacy of SARS-CoV-2 S-mediated cell–cell fusion (Figure 4F, top). Conversely, the assay based on B.1 S showed that the S375F mutation (spikes 16 and 18–20) decreased pseudovirus infectivity (Figure 4B, bottom), S cleavage efficacy (Figures 4C and 4D, bottom) and fusion activity (Figure 4F, bottom). These results suggest that the S375F mutation in Omicron S is responsible for the decreased S cleavage efficacy in producer cells and the attenuated fusogenicity observed. However, the S371L/S373P/S375F mutations did not affect sensitivity to the antiviral humoral immunity elicited by vaccination and infection (Figure S3), suggesting that the S375F mutation is not associated with the immune resistant phenotype of Omicron.

**Figure 5:**
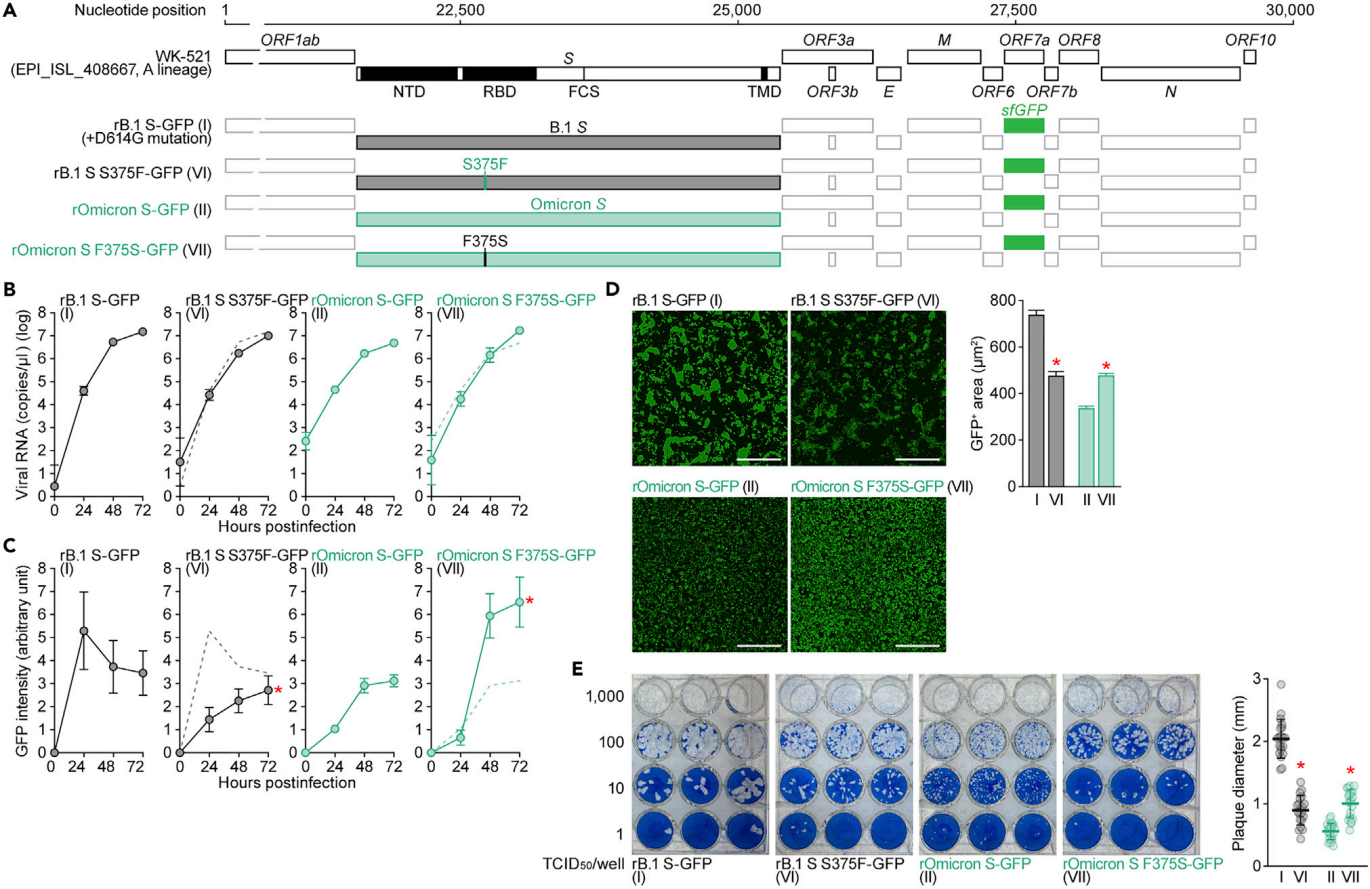
Effect of the S S375F mutation on viral growth dynamics (A) Scheme of the S-chimeric recombinant SARS-CoV-2 used in this study. The numbers in parentheses are identical to those in Figures 5B–5E. (B–D) SARS-CoV-2 infection. VeroE6/TMPRSS2 cells were infected with a series of S-chimeric recombinant SARS-CoV-2 (summarized in A) at an m.o.i. 0.01. The viral RNA in the supernatant (B) and GFP intensity (C) were measured routinely. Note that the yaxes of the graphs shown in B are log scales. The results for the respective parental S are shown in other panels as broken green lines. Assays were performed in quadruplicate (B and C). (D) Syncytium formation. Left, GFP-positive area at 48 h.p.i. Scale bar, 500 μm. Right, summarized results. I, n = 6,483 cells; VI, n = 2,780 cells; II, n = 5,393 cells; and VII, 12,857 cells. The results for B.1-GFP (virus I) and Omicron-GFP (virus II) in C and D (right) are identical to those shown in Figures 1I and 1J (right). Representative images are shown in Figure S1. (E) Plaque assay. Left, representative figures.Right, summary of the plaque diameters (20 plaques per virus). Each dot indicates the result of an individual plaque. Data are expressed as the mean with SD (B, C, and E) or the median with 95% CI (D). In B and C, statistically significant differences (∗FWERs<0.05) versus Omicron-GFP (virus II) through timepoints were determined by multiple regression. FWERs were calculated using the Holm method. In D and E, statistically significant differences (∗p <0.05) versus Omicron-GFP (virus II) were determined by a two-sided Mann–Whitney U test. See also Figure S1.

To further assess the impact of the S375F mutation, we generated two additional recombinant chimeric SARS-CoV-2 strains, B.1 S S375F-GFP (virus VI) and Omicron S F375S-GFP (virus VII) (Figure 5A). Although the mutation at position 375 of the S protein did not affect the viral RNA load in the culture supernatant of infected VeroE6/TMPRSS2 cells (Figure 5B), the GFP intensity in infected VeroE6/TMPRSS2 cells was significantly altered by this mutation: the S375F mutation in the B.1 S backbone decreased the GFP intensity, whereas the F375S mutation in the Omicron S backbone increased the intensity (Figures 5C and S1). In addition, quantitative fluorescence microscopy showed that the GFP-positive area of B.1 S S375F-GFP (virus VI) was significantly lower than that of parental B.1 S-GFP (virus I); however, that of Omicron S F375S-GFP (virus VII) was significantly higher than that of parental Omicron S-GFP (virus II) (Figure 5D). Moreover, plaque assays showed that the plaques formed by infection with B.1 S S375F-GFP (virus VI) were significantly smaller than those formed by B.1 S-GFP (virus I); conversely, plaque size was increased by the presence of the F375S mutation in Omicron S (Figure 5E). Altogether, these results suggest that the S375F mutation in the Omicron S protein determines the major virological characteristics (i.e., decreased S1/S2 cleavage efficacy, decreased fusogenicity, and decreased ACE2 binding affinity) of Omicron.

### F375-H505 pi-pi interaction contributes to the decreased cleavage efficacy and fusogenicity of Omicron S

**Figure 6:**
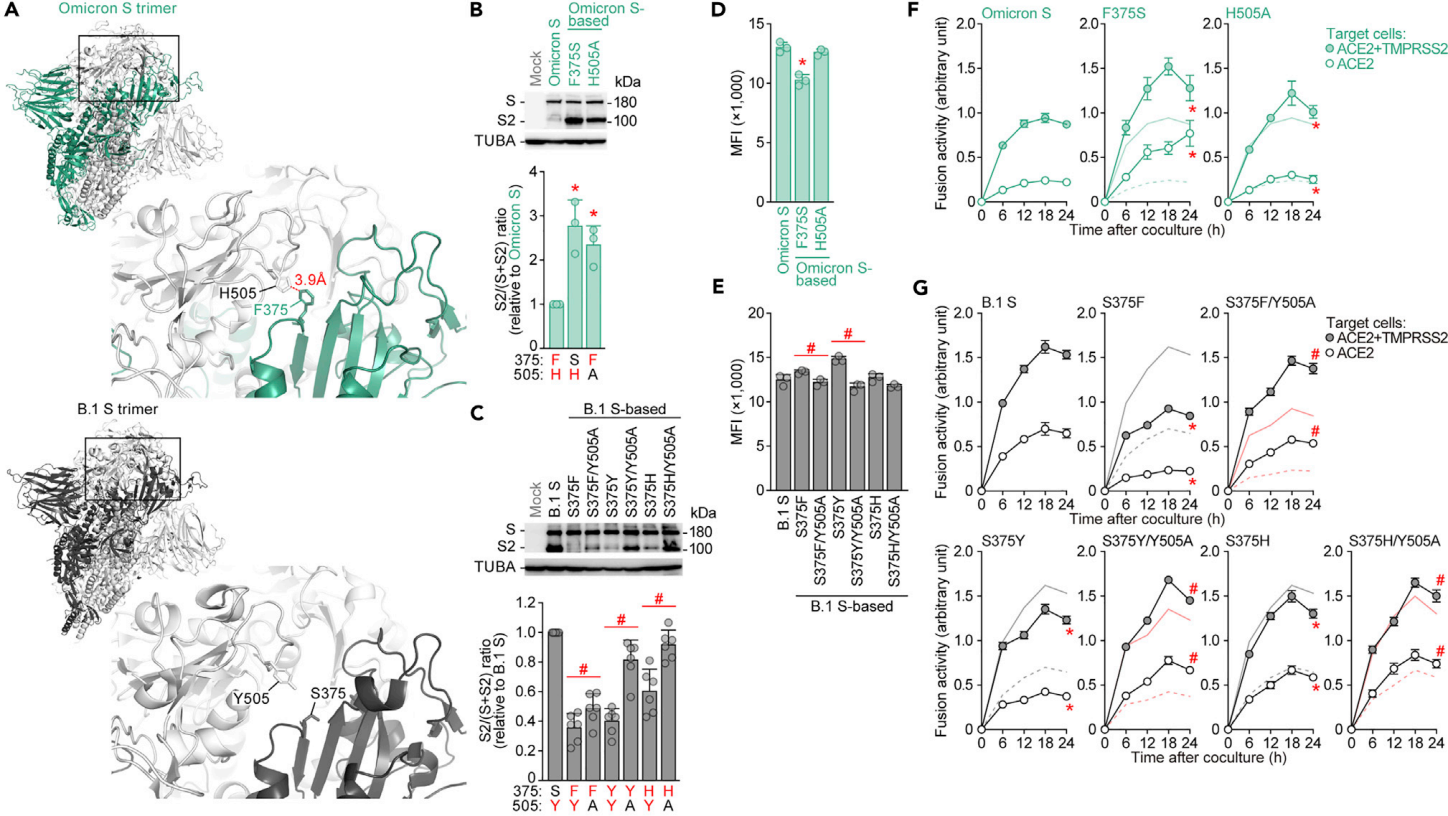
Effect of the pi-pi interaction between 375F and 505H (A) Structural insights into the SARS-CoV-2 S trimer. Top, the structure of the Omicron S trimer (PDB: 7T9J)^29^ reconstructed as described in the STAR Methods. Bottom, cryo-EM structure of the B.1 S trimer (PDB: 7KRQ).^28^ The regions indicated in squared are enlarged in the bottom right panels. In the enlarged panels, the residues at position 375 [F in an Omicron S monomer indicated in green (top); S in a B.1 S monomer indicated in black (bottom)] and 505 [H in an Omicron S monomer indicated in white (top); Y in a B.1 S monomer indicated in white (bottom)] are shown. The putative pi-pi interaction between F375 and H505 in the Omicron S trimer is indicated in red (3.9 Å) (B and C) Western blot. Representative blots of S-expressing cells (top) and quantified band intensity (the ratio of S2 to the full-length S plus S2 proteins) (bottom) are shown. In the bottom panels, the residues at positions 375 and 505 are indicated, and aromatic residues (F, H or Y) are indicated in red. Uncropped blots are shown in Figure S4. (D and E) Flow cytometry. The summarized results of the surface S expression are shown. (F and G) SARS-CoV-2 S-based fusion assay. The fusion activity was measured as described in the STAR Methods, and fusion activity (arbitrary units) is shown. For the target cells, HEK293 cells expressing ACE2 and TMPRSS2 (filled) and HEK293 cells expressing ACE2 (open) were used. In F, normal lines, Omicron S with HEK293-ACE2/TMPRSS2 cells; broken lines, Omicron S with HEK293-ACE2 cells. In the panels of S375F, S375Y and S375H in G, normal black lines, B.1 S using HEK293-ACE2/TMPRSS2 cells; broken black lines, B.1 S using HEK293-ACE2 cells; normal red lines. In the panels for S375F/Y505A, S375Y/Y505A and S375H/Y505A in G, normal red line, the result for the respective mutant without the Y505A mutation using HEK293-ACE2/TMPRSS2 cells; broken red line, the result for the respective mutant without the Y505A mutation using HEK293-ACE2 cells. Data are expressed as the mean with SD (B–G). Assays were performed in triplicate (B, D–G) or sextuplicate (C). In B–E, each dot indicates the result of an individual replicate. Statistically significant differences versus Omicron S (∗p <0.05) and between the mutant with and without the Y505A mutation (#p <0.05) were determined by two-sided paired *t* test (B and C) or two-sided Student’s *t* test (D and E). In F and G, statistically significant differences versus Omicron S (∗FWERs<0.05) or the mutant without the Y505A mutation (#FWERs<0.05) through timepoints were determined by multiple regression. FWERs were calculated using the Holm method. See also Figure S4.

Here, we experimentally demonstrate that the S375F mutation attenuates the cleavage efficacy and fusogenicity of Omicron S (Figures 4 and 5). In addition, molecular phylogenetic analysis suggested that the emergence of this mutation was closely associated with the explosive growth of Omicron in the human population (Figures 3C and 3D). Nevertheless, it remains unclear how the S375F mutation contributes to the decrease in cleavage efficacy and fusogenicity of Omicron S at the molecular level. We addressed this question using a structural biology approach. As shown in Figure 6A (top), we predicted that the F375 residue in a fully closed Omicron S trimer could form a pi-pi interaction, a sort of dispersion via van der Waals forces between aromatic residues,^30^ with the H505 residue in another S protein of the same trimer. Importantly, the cryo-EM structure of the Omicron BA.1 S protein has been determined.^31^ The result demonstrated that the interprotomer interaction mediated by the F375 and H505 residues of Omicron S causes the S trimer conformation to be more rigid and leads to less cleavage efficacy, supporting our prediction. Because residue 375 in the B.1.1 S protein is a serine, the pi-pi interaction cannot be formed (Figure 6A, bottom). To address the hypothesis that the F375-H505-mediated interprotomer pi-pi interaction contributes to the decreased cleavage efficacy and fusogenicity of Omicron S, we prepared the Omicron S H505A mutant, in which an aromatic side chain at position 505 is disrupted. Western blot analysis showed that the cleavage efficacy of Omicron S was increased by the insertion of the H505A mutation (Figure 6B). To further test this possibility, the residues at position 375 of B.1 S were substituted with amino acids bearing aromatic side chains (i.e., F, Y and H). Similar to the S375F mutant, the B.1 S mutants bearing the S375Y or S375H mutation showed decreased S protein cleavage efficacy (Figure 6C). These results further suggest that the interprotomer pi-pi interaction is formed between Y505 and S375F/Y/H. Moreover, insertion of the Y505A mutation in B.1 S bearing the S375F/Y/H mutation (i.e., disruption of the aromatic residue at position 505) rescued the S cleavage efficacy (Figure 6C).

Finally, we verified the impact of the interprotein pi-pi interaction on S-mediated fusogenicity. The Omicron S F375S mutant exhibited decreased surface expression, but the H505A mutation did not (Figure 6D). In the case of the B.1 S-based mutants, the Y505A mutation decreased surface expression levels when the S375F/Y mutations were also present (Figure 6E). Corresponding to western blot results (Figure 6B), disruption of the pi-pi interaction by F375S and H505A in Omicron S significantly increased fusion activity (Figure 6F). Moreover, in the case of the B.1 S-based mutant, substitution of residue 375 with an aromatic residue (F, Y or H) significantly reduced fusion activity (Figure 6G). However, when the Y505A substitution was present in the S375F/Y/H mutants, disrupting the aromatic residue at position 505, fusion activity was significantly increased (Figure 6G). Altogether, our results suggest that the interprotomer pi-pi interaction mediated by the aromatic residues at positions 375 and 505 of the S protein contributes to the decreased cleavage efficacy and fusogenicity of Omicron S.

## Discussion

In the present study, we performed multiscale investigations to unveil the virological characteristics of the S protein of the SARS-CoV-2 Omicron variant, including (1) profound immune resistance, (2) decreased cleavage efficacy in cells, (3) poor fusogenicity, and (4) reduced ACE2 binding affinity. By using pseudoviruses, a yeast surface display system and the chimeric recombinant SARS-CoV-2 generated by reverse genetics, we showed that the RBD of Omicron S is responsible for these four virological features of this variant. In particular, the S375F mutation in the RBD of Omicron S is one of the most critical mutations that determine three of the four major virological properties of Omicron: decreased affinity to ACE2, attenuated efficacy of S cleavage, and reduced fusogenicity. Moreover, molecular phylogenetic analysis provided evidence suggesting that the acquisition of the S375F mutation was closely related to the onset of the explosive spread of Omicron in the human population (Figure 3C). Furthermore, experiments based on structural biology revealed that the pi-pi interaction mediated by residues F375 and H505 is responsible for the observed decreased cleavage efficacy in cells and fusogenicity.

We and others demonstrate that the Omicron S RBD shows reduced binding affinity to human ACE2.^14^^,^^23^ In this study, our mutagenesis experiment revealed that the S375F, G496S and Y505H substitutions are responsible for this reduced binding affinity of the Omicron S RBD to human ACE2 (Figure 3B). Considering the importance of ACE2 binding in viral replication, it is intriguing how the Omicron variant acquired high transmissibility with decreased ACE2 binding. It may be reasonable to speculate that evasion from the preexisting immunity induced by previous infection or vaccination was the priority for the evolution of Omicron.

We revealed that the nascent pi-pi interaction of the Omicron S trimer is established by the F375 and H505 residues and characterizes Omicron S. After the initial submission of this study, structural analysis by cryo-electron microscopy (cryo-EM) showed that the interprotomer interaction mediated by the F375 and H505 residues of Omicron S firms the S trimer conformation and leads to reduced cleavage efficacy,^31^supporting our experimental results in this study. Because the Y505H mutation was already present in the ancestral Omicron sequences, our results suggest that acquisition of the S375F mutation during the evolution of Omicron resulted in attenuated S cleavage efficacy and fusogenicity in SARS-CoV-2 S protein, which led to the explosive spread of Omicron in the human population. The S375F mutation is highly conserved in the Omicron lineage and has not been detected in the other SARS-CoV-2 variants. However, our data suggest that substitution of residues possessing an aromatic ring, such as phenylalanine, tyrosine and histidine, at residue 375 may confer Omicron-like properties. Therefore, the emergence of SARS-CoV-2 variants bearing such substitutions at residue 375 should be considered a potential risk for health of the global population.

Our previous studies suggested a close association between viral fusogenicity and pathogenicity.^22^^,^^23^^,^^32^ For example, Omicron S is less susceptible to cleavage than parental B.1.1 S harboring the D614G mutation.^18^^,^^22^ This decreased S1/S2 cleavage is associated with a reduction in the fusogenicity of Omicron S and attenuates the pathogenicity of Omicron variant.^22^ Here, we demonstrate that S cleavage efficacy and fusogenicity are determined by S375F mutation in the RBD of Omicron S (Figures 1C, 1F, 4C and 4F). Therefore, it is likely that acquisition of the S375F mutation in the S protein may, at least partially, contribute to the attenuated pathogenicity of the Omicron variant. Further investigation will be required to determine whether the S375F mutation is critical for viral pathogenicity because this mutation is present among more than 30 changes.

Here, we show the importance of the S375F mutation to the major virological properties of Omicron S, particularly its decreased cleavage efficacy, poor fusogenicity, and reduced ACE2 binding affinity. However, the following issues remain to be fully elucidated. First, although we showed that the S375F mutation determines a part of the virological features of Omicron S, it remains unclear which mutations in Omicron S determine its pronounced immune resistance. We showed that the RBD of Omicron S is closely associated with its resistance to the humoral immunity induced by vaccination and natural SARS-CoV-2 infection (Figure 2), yet there are dozen substitutions in the Omicron S RBD (Figure 3B). Therefore, it would be reasonable to assume that multiple substitutions in the RBD cooperatively contribute to the profound immune resistance of Omicron S. Second, in addition to the Omicron BA.1 variant that we focused on this study, a variety of Omicron subvariants, such as BA.2 and BA.5, have emerged, and these subvariants also bear the S375F mutation. However, we have recently shown that the fusogenicity of BA.2 S is significantly higher than that of BA.1 S.^23^ Together with the results of this study, these observations suggest that BA.2 S has acquired certain compensatory mutation(s) that increase fusion efficacy. Further investigations will be needed to unveil the full evolutionary history of the Omicron lineage. Furthermore, the question of why acquisition of the S375F mutation caused explosive spread despite reduced infectivity in tissue culture, S cleavage efficacy and fusogenicity also needs to be elucidated in detail by further studies.

In summary, our multiscale investigations reveal that the major virological characteristics of Omicron S, namely, attenuated S cleavage efficacy, attenuated fusogenicity, and reduced ACE2 binding affinity, are determined by one specific mutation, S375F, in the RBD. Assays based on structural biology revealed that the pi-pi interaction mediated by residues F375 and H505 is responsible for the observed attenuated S cleavage efficacy and fusogenicity. Furthermore, the molecular phylogenetic analysis suggested that acquisition of the S375F mutation was closely associated with the massive spread of Omicron in the human population. Altogether, our results suggest that acquisition of the S375F mutation was a crucial event for the emergence of a highly transmissible SARS-CoV-2 variant, Omicron.

### Limitations of the study

In this study, we used lentivirus-based pseudovirus to examine impact mutations on S packaging; thus, the phenotype observed in this study may differ from the S2 incorporation occurring in authentic SARS-CoV-2 virions. However, our recent finding with authentic SARS-CoV-2 virions demonstrated that the S2 incorporation pattern of BA.1 was significantly lower than that of the Delta variant,^18^ reproducing the low S2 incorporation of BA.1 S observed in this study. Therefore, these data suggest that lentivirus-based pseudovirus can be used to examine the S2 incorporation pattern of authentic SARS-CoV-2 virions. Further investigations, such as the identification of S protein incorporation in lentivirus-based pseudoviruses, are required to solve this issue.

In addition, it is reported that the Omicron variants deposited early contain artifactual reversions possibly derived from contamination of non-Omicron (mainly Delta) variants due to the low affinity of primers for sequencing.^33^^,^^34^ Although such low-quality Omicron genomes were removed in this analysis (see STAR Methods), we cannot exclude the possibility that some genomes may contain artifactual reversions, which would affect the phylogenetic results.

## Consortia

The Genotype-to-Phenotype Japan (G2P-Japan) Consortium, Keita Matsuno, Naganori Nao, Hirofumi Sawa, Mai Kishimoto, Shinya Tanaka, Masumi Tsuda, Lei Wang, Yoshikata Oda, Marie Kato, Zannatul Ferdous, Hiromi Mouri, Kenji Shishido, Takasuke Fukuhara, Tomokazu Tamura, Rigel Suzuki, Hayato Ito, Naoko Misawa, Shigeru Fujita, Mai Suganami, Mika Chiba, Ryo Yoshimura, Yasuhiro Kazuma, Ryosuke Nomura, Yoshihito Horisawa, Yusuke Tashiro, Yugo Kawai, Ryoko Kawabata, MST Monira Begum, Otowa Takahashi, Kimiko Ichihara, Chihiro Motozono, and Maya Shofa.

## STAR★Methods

### Key resources table

**Table:** 

REAGENT or RESOURCE	SOURCE	IDENTIFIER
**Antibodies**		

Rabbit anti-SARS-CoV-2 S S1/S2 polyclonal antibody (1:100 for FACS)	Thermo Fisher Scientific	Cat# PA5-112048; RRID: AB_2866784
Normal rabbit IgG (1:100 for FACS)	SouthernBiotech	Cat# 0111-01; RRID: AB_2732899
APC-conjugated goat anti-rabbit IgG polyclonal antibody (1:50 for FACS)	Jackson ImmunoResearch	Cat# 111-136-144; RRID: AB_2337987
Mouse anti-SARS-CoV-2 S monoclonal antibody (clone 1A9) (1:10,000 for immunoblotting)	GeneTex	Cat# GTX632604; RRID: AB_2864418
Mouse anti-HIV-1 p24 monoclonal antibody (clone 183-H12-5C) (1:2,000 for immunoblotting)	NIH HIV Reagent Program	Cat# ARP-3537; RRID: AB_2819250
Rabbit anti-beta actin (ACTB) monoclonal antibody (clone 13E5) (1:5,000 for immunoblotting)	Cell Signaling	Cat# 4970; RRID: AB_2223172
Mouse anti-tubulin (TUBA) monoclonal antibody (clone DM1A) (1:10,000 for immunoblotting)	Sigma-Aldrich	Cat# T9026; RRID: AB_477593
HRP-conjugated horse anti-mouse IgG antibody (1:2,000 for immunoblotting)	Cell Signaling	Cat# 7076S; RRID: AB_330924
HRP-conjugated donkey anti-rabbit IgG polyclonal antibody (1:10,000 for immunoblotting)	Jackson ImmunoResearch	Cat# 711-035-152; RRID: AB_10015282
HRP-conjugated donkey anti-mouse IgG polyclonal antibody (1:10,000 for immunoblotting)	Jackson ImmunoResearch	Cat# 715-035-150; RRID: AB_2340770

**Bacterial and virus strains**		

Recombinant SARS-CoV-2, rB.1 S-GFP	(Saito et al.^32^; Yamasoba et al.^23^)	N/A
Recombinant SARS-CoV-2, rOmicron S-GFP	(Yamasoba et al.^23^)	N/A
Recombinant SARS-CoV-2, rOmicron S/B.1 S_NTD-GFP	This study	N/A
Recombinant SARS-CoV-2, rOmicron S/B.1 S_RBD-GFP	This study	N/A
Recombinant SARS-CoV-2, rOmicron S/B.1 S_NTD+RBD-GFP	This study	N/A
Recombinant SARS-CoV-2, rB.1 S S375F-GFP	This study	N/A
Recombinant SARS-CoV-2, rOmicron S F375S-GFP	This study	N/A

**Biological samples**		

Human sera (see details in Table S1)	This study	N/A

**Chemicals, peptides, and recombinant proteins**		

TransIT-LT1 Transfection Reagent	Takara	Cat# MIR2300
TransIT-293 Transfection Reagent	Takara	Cat# MIR2700
Recombinant RNase inhibitor	Takara	Cat# 2313B
Carboxymethyl cellulose	Wako	Cat# 039-01335
4% paraformaldehyde phosphate buffer solution	Nacalai Tesque	Cat# 09154-85
Methylene blue	Nacalai Tesque	Cat# 22412-14
Fetal bovine serum	Sigma-Aldrich	Cat# 172012-500ML
Penicillin-streptomycin	Sigma-Aldrich	Cat# P4333-100ML
DMEM (high glucose)	Sigma-Aldrich	Cat# 6429-500ML
DMEM (low glucose)	Wako	Cat# 041-29775
Expi293 expression medium	Thermo Fisher Scientific	Cat# A1435101
Ham’s F-12K	Wako	Cat# 080-08565
Puromycin	Sigma-Aldrich	Cat# P9620-10ML
Blasticidin	InvivoGen	Cat# ant-bl-1
G418	Nacalai Tesque	Cat# G8168-10ML
KpnI	New England Biolab	Cat# R0142S
NotI	New England Biolab	Cat# R1089S
PEI Max	Polysciences	Cat# 24765-1
Nonidet P40 substitute	Nacalai Tesque	Cat# 18558-54
Protease inhibitor cocktail	Nacalai Tesque	Cat# 03969-21
Protein assay dye	Bio-Rad	Cat# 5000006
4 × NuPAGE LDS sample buffer	Thermo Fisher Scientific	Cat# NP0007
Doxycycline	Takara	Cat# 1311N
TURBO DNase	Thermo Fisher Scientific	Cat# AM2238
Triton X-100	Nacalai Tesque	Cat# 35501-15
EnduRen live cell substrate	Promega	Cat# E6481
Glycerol	Nacalai Tesque	Cat# 17018-25
Soluble human ACE2 (residues 18–740)	(Yamasoba et al.^23^)	N/A
SARS-CoV-2 B.1 S RBD	(Kimura et al.^27^; Motozono et al.^24^; Yamasoba et al.^23^)	N/A
SARS-CoV-2 B.1 S RBD G339D	This study	N/A
SARS-CoV-2 B.1 S RBD S371L	This study	N/A
SARS-CoV-2 B.1 S RBD S373P	This study	N/A
SARS-CoV-2 B.1 S RBD S375F	This study	N/A
SARS-CoV-2 B.1 S RBD S371L/S373P/S375F	This study	N/A
SARS-CoV-2 B.1 S RBD N440K	This study	N/A
SARS-CoV-2 B.1 S RBD G446S	This study	N/A
SARS-CoV-2 B.1 S RBD S477N	This study	N/A
SARS-CoV-2 B.1 S RBD E484A	This study	N/A
SARS-CoV-2 B.1 S RBD Q493R	This study	N/A
SARS-CoV-2 B.1 S RBD G496S	This study	N/A
SARS-CoV-2 B.1 S RBD Q498R	This study	N/A
SARS-CoV-2 B.1 S RBD Y505H	This study	N/A
SARS-CoV-2 Omicron S RBD	(Dejnirattisai et al.^14^; Yamasoba et al.^23^)	N/A
SARS-CoV-2 Omicron S RBD L371S	This study	N/A
SARS-CoV-2 Omicron S RBD P373S	This study	N/A
SARS-CoV-2 Omicron S RBD F375S	This study	N/A
SARS-CoV-2 Omicron S RBD L371S/P373S/F375S	This study	N/A
Bilirubin	Sigma-Aldrich	Cat# 14370-1G
CF®640R succinimidyl ester	Biotium	Cat# 92108

**Critical commercial assays**		

QIAamp viral RNA mini kit	Qiagen	Cat# 52906
NEB next ultra RNA library prep kit for Illumina	New England Biolabs	Cat# E7530
MiSeq reagent kit v3	Illumina	Cat# MS-102-3001
OneStep TB Green PrimeScript PLUS RT-PCR kit	Takara	Cat# RR096A
SARS-CoV-2 direct detection RT-qPCR kit	Takara	Cat# RC300A
Nano Glo HiBiT lytic detection system	Promega	Cat# N3040
KAPA HiFi HotStart ReadyMix kit	Roche	Cat# KK2601
PrimeSTAR GXL DNA polymerase	Takara	Cat# R050A
Bright-Glo luciferase assay system	Promega	Cat# E2620
One-Glo luciferase assay system	Promega	Cat# E6130
SuperSignal West Femto Maximum Sensitivity Substrate	Thermo Fisher Scientific	Cat# 34095
SuperSignal West Atto Ultimate Sensitivity Substrate	Thermo Fisher Scientific	Cat# A38554
Western BLoT UltraSensitive HRP Substrate	Takara	Cat# T7104A
GENEART site-directed mutagenesis system	Thermo Fisher Scientific	Cat# A13312
ACE2 activity assay kit	SensoLyte	Cat# AS-72086

**Deposited data**		

Viral genome sequencingdata of working viral stocks (see Table S4)	DDBJ Sequence Read Archive	Accession number: PRJDB13805

**Experimental models: Cell lines**		

Human: HEK293T cells	ATCC	CRL-3216
Human: HEK293 cells	ATCC	CRL-1573
Human: HEK293-C34 cells	(Torii et al.^25^)	N/A
Human: Expi293F cells	Thermo Fisher Scientific	Cat# A14527
Human: HOS-ACE2/TMPRSS2 cells	(Ferreira et al.^35^; Ozono et al.^36^)	N/A
Human: A549-ACE2 cells	(Motozono et al.^24^)	N/A
African green monkey (*Chlorocebus sabaeus*): VeroE6/TMPRSS2 cells	JCRB Cell Bank (Matsuyama et al.^37^)	JCRB1819
Yeast (*Saccharomyces cerevisiae*): strain EBY100	ATCC	MYA-4941

**Oligonucleotides**		

Primers for the construction of plasmids expressing the codon-optimized S proteins of a series of SAR-CoV-2 S mutants and chimeras, see Table S3	This study	N/A
Primers for SARS-CoV-2 reverse genetics, see Table S3	This study	N/A
RT-qPCR, forward: AGC CTC TTC TCG TTC CTC ATC AC	(Meng et al.^18^; Motozono et al.^24^; Saito et al.^32^; Suzuki et al.^22^; Yamasoba et al.^23^)	N/A
RT-qPCR, reverse: CCG CCA TTG CCA GCC ATT C	(Meng et al.^18^; Motozono et al.^24^; Saito et al.^32^; Suzuki et al.^22^; Yamasoba et al.^23^)	N/A
Primers for the construction of yeast-optimized SARS-CoV-2 B.1 and Omicron S RBD expression plasmid, see Table S3	This study	N/A

**Recombinant DNA**		

Plasmid: pCAGGS	(Niwa et al.^38^)	N/A
Plasmid: psPAX2-IN/HiBiT	(Ozono et al.^39^)	N/A
Plasmid: pWPI-Luc2	(Ozono et al.^39^)	N/A
Plasmid: pC-ACE2	(Ozono et al.^36^)	N/A
Plasmid: pC-TMPRSS2	(Ozono et al.^36^)	N/A
Plasmid: pJYDC1	Addgene	Cat# 162458
Plasmid: pDSP_1-7_	(Kondo et al., 2011)	N/A
Plasmid: pDSP_8-11_	(Kondo et al., 2011)	N/A
Plasmid: pC-B.1 S	(Motozono et al.^24^; Ozono et al., 2021^36^)	N/A
Plasmid: pC-Delta S (B.1.617.2 S)	(Kimura et al.^27^; Saito et al.^32^)	N/A
Plasmid: pC-Omicron S (BA.1 S)	(Meng et al.^18^; Suzuki et al.^22^)	N/A
Plasmid: pC-Omicron S/B.1 S_NTD	This study	N/A
Plasmid: pC-Omicron S/B.1 S_RBD	This study	N/A
Plasmid: pC-Omicron S/B.1 S_RBD	This study	N/A
Plasmid: pC-Omicron S/B.1 S_NTD+RBD	This study	N/A
Plasmid: pC-B.1 S/Omicron S_RBD	This study	N/A
Plasmid: pC-Omicron S L371S	This study	N/A
Plasmid: pC-Omicron S P373S	This study	N/A
Plasmid: pC-Omicron S F375S	This study	N/A
Plasmid: pC-Omicron S L371S/P373S	This study	N/A
Plasmid: pC-Omicron S P373S/F375S	This study	N/A
Plasmid: pC-Omicron S L371S/F375S	This study	N/A
Plasmid: pC-Omicron S L371S/P373S/F375S	This study	N/A
Plasmid: pC-B.1 S S371L	This study	N/A
Plasmid: pC-B.1 S S373P	This study	N/A
Plasmid: pC-B.1 S S375F	This study	N/A
Plasmid: pC-B.1 S S375F/Y505A	This study	N/A
Plasmid: pC-B.1 S S371L/S373P	This study	N/A
Plasmid: pC-B.1 S S373P/S375F	This study	N/A
Plasmid: pC-B.1 S S371L/S375F	This study	N/A
Plasmid: pC-B.1 S S371L/S373P/S375F	This study	N/A
Plasmid: pC-B.1 S S375Y	This study	N/A
Plasmid: pC-B.1 S S375Y/Y505A	This study	N/A
Plasmid: pC-B.1 S S375H	This study	N/A
Plasmid: pC-B.1 S S375H/Y505A	This study	N/A
Plasmid: pC-B.1 S S375F/Y505A	This study	N/A
Plasmid: pC-B.1 S S375Y/Y505A	This study	N/A
Plasmid: pC-B.1 S S375H/Y505A	This study	N/A
Plasmid: pC-Omicron S H505A	This study	N/A

**Software and algorithms**		

fastp v0.21.0	(Chen et al.^40^)	https://github.com/OpenGene/fastp
BWA-MEM v0.7.17	(Li and Durbin,^41^)	http://bio-bwa.sourceforge.net
SAMtools v1.9	(Li et al.^42^)	http://www.htslib.org
snpEff v5.0e	(Cingolani et al.^43^)	http://pcingola.github.io/SnpEff
RDP4 v4.101	(Martin et al.^44^)	http://web.cbio.uct.ac.za/∼darren/rdp.html
MAFFT suite v7.407	(Katoh and Standley,^45^)	https://mafft.cbrc.jp/alignment/software
BEAST v1.10.4	(Suchard et al.^46^)	https://beast.community
FigTree v1.4.4	http://tree.bio.ed.ac.uk/software/figtree/	http://tree.bio.ed.ac.uk/software/figtree/
Tracer v1.7.1	(Rambaut et al.^47^)	https://beast.community/tracer.html
R v4.1.3	The R Foundation	https://www.r-project.org/
Sequencher v5.1 software	Gene Codes Corporation	N/A
Prism 9 software v9.1.1	GraphPad Software	https://www.graphpad.com/scientific-software/prism/
Fiji software v2.2.0	ImageJ	https://fiji.sc
Image Studio Lite v5.2	LI-COR Biosciences	https://www.licor.com/bio/image-studio/
FlowJo software v10.7.1	BD Biosciences	https://www.flowjo.com/solutions/flowjo
Python v3.7	Python Software Foundation	https://www.python.org
PyMOL molecular graphics system v2.5.0	Schrödinger	https://pymol.org/2/
BZ-X800 analyzer software	Keyence	N/A
Photoshop 2021 v22.4.1	Adobe	N/A

**Other**		

Centro XS3 LB960	Berthhold Technologies	N/A
GloMax explorer multimode microplate reader 3500	Promega	N/A
96-well black plate	PerkinElmer	Cat# 6005225
FACS Canto II	BD Biosciences	N/A
GISAID database	(Khare et al., 2021)	https://doi.org/10.55876/gis8.221004su
QuantStudio 3 Real-Time PCR system	Thermo Fisher Scientific	N/A
Thermal Cycler Dice Real Time System III	Takara	N/A
CFX Connect Real-Time PCR Detection system	Bio-Rad	N/A
Eco Real-Time PCR System	Illumina	N/A
qTOWER3 G Real-Time System	Analytik Jena	N/A
7500 Real-Time PCR System	Thermo Fisher Scientific	N/A
Amersham Imager 600	GE Healthcare	N/A
iBright FL1500 Imaging System	Thermo Fisher Scientific	N/A
All-in-One Fluorescence Microscope BZ-X800	Keyence	N/A
HisTrap Fast Flow column	Cytiva	Cat# 17-5255-01
Superdex 200 16/600	Cytiva	Cat# 28-9893-35
ÄKTA pure chromatography system	Cytiva	N/A
Tycho NT.6 system	NanoTemper	N/A
FACS S3e Cell Sorter device	Bio-Rad	N/A

### Resource availability

#### Lead contact

Further information and requests for resources and reagents should be directed to and will be fulfilled by the lead contact, Kei Sato (keisato@g.ecc.u-tokyo.ac.jp).

#### Materials availability

All unique reagents generated in this study are listed in the key resources table and available from the lead contact with a completed Materials Transfer Agreement.

### Experimental model and subject details

#### Ethics statement

All protocols involving specimens from human subjects recruited at Kyoto University and Kuramochi Clinic Interpark were reviewed and approved by the Institutional Review Boards of Kyoto University (approval ID: G1309) and Kuramochi Clinic Interpark (approval ID: G2021-004). All human subjects provided written informed consent. All protocols for the use of human specimens were reviewed and approved by the Institutional Review Boards of The Institute of Medical Science, The University of Tokyo (approval IDs: 2021-1-0416 and 2021-18-0617), Kyoto University (approval ID: G0697), Kumamoto University (approval IDs: 2066 and 2074), and University of Miyazaki (approval ID: O-1021).

#### Human serum collection

Vaccine sera were collected from eleven vaccinees four weeks after their second vaccination with the BNT162b2 (Pfizer/BioNTech) vaccine (average age: 35, range: 29–56, 18% male) and sixteen vaccinees four weeks after their second mRNA-1273 (Moderna) vaccine (average age: 27, range: 20–47, 38% male).

Convalescent sera were collected from vaccine-naïve individuals who had been infected with the Delta variant (n = 10; average age: 47, range: 22–63, 70% male). To identify the SARS-CoV-2 variants infecting patients, saliva was collected from COVID-19 patients during infection onset, and RNA was extracted using a QIAamp viral RNA mini kit (Qiagen, Cat# 52906) according to the manufacturer’s protocol. To identify the Delta variants, viral genome sequencing was performed as previously described.^18^ For details, see the "viral genome sequencing" section below. Sera collected from twelve convalescents during the early pandemic (until May 2020) (average age: 71, range: 52–92, 8% male) were purchased from RayBiotech. Sera were inactivated at 56°C for 30 min and stored at −80°C until use. The details of the sera used in this study are summarized in Table S1.

#### Cell culture

HEK293T cells (a human embryonic kidney cell line; ATCC, CRL-3216), HEK293 cells (a human embryonic kidney cell line; ATCC CRL-1573), and HOS-ACE2/TMPRSS2 cells, HOS cells (a human osteosarcoma cell line; ATCC CRL- 1543) stably expressing human ACE2 and TMPRSS2^35^^,^^36^ were maintained in Dulbecco’s modified Eagle’s medium (DMEM) (high glucose) (Sigma-Aldrich, Cat# 6429-500ML) containing 10% fetal bovine serum (FBS) and 1% penicillin-streptomycin (PS) (Sigma-Aldrich, Cat# P4333-100ML). HEK293-C34 cells, *IFNAR1* KO HEK293 cells expressing human ACE2 and TMPRSS2 by doxycycline treatment,^25^ were maintained in DMEM (high glucose) containing 10% FBS, 10 μg/mL blasticidin (InvivoGen, Cat# ant-bl-1) and 1% PS. VeroE6/TMPRSS2 cells (VeroE6 cells stably expressing human TMPRSS2; JCRB1819)^37^ were maintained in DMEM (low glucose) (Wako, Cat# 041-29775) containing 10% FBS, G418 (1 mg/mL; Nacalai Tesque, Cat# G8168-10ML) and 1% PS. Expi293F cells (Thermo Fisher Scientific, Cat# A14527) were maintained in Expi293 expression medium (Thermo Fisher Scientific, Cat# A1435101). A549-ACE2 cells, A549 cells (a human lung epithelial cell line; ATCC CCL-185) stably expressing human ACE2^24^ were maintained in Ham’s F-12K (Wako, Cat# 080-08565) containing 10% FBS, puromycin (1 μg/mL; Sigma-Aldrich, Cat# P9620-10ML) and 1% PS.

### Method details

#### Viral genome sequencing

The virus sequences were verified by viral RNA-sequencing analysis. Viral RNA was extracted using a QIAamp viral RNA mini kit (Qiagen, Cat# 52906). The sequencing library employed for total RNA sequencing was prepared using the NEB Next Ultra RNA Library Prep Kit for Illumina (New England Biolabs, Cat# E7530). Paired-end 76-bp sequencing was performed using a MiSeq system (Illumina) with MiSeq reagent kit v3 (Illumina, Cat# MS-102-3001). Sequencing reads were trimmed using fastp v0.21.0^40^ and subsequently mapped to the viral genome sequences of a lineage A isolate (strain WK-521; GISAID ID: EPI_ISL_408667)^37^ using BWA-MEM v0.7.17.^41^ Variant calling, filtering, and annotation were performed using SAMtools v1.9^42^ and snpEff v5.0e.^43^

#### Molecular phylogenetic analyses

The SARS-CoV-2 genomes and annotation information used in this study were downloaded from the GISAID EpiCoV database (https://www.gisaid.org/) on January 8, 2022 (6,780,682 sequences). A total of 204,375 Omicron BA.1 variants were obtained, which included 1,074 B.1.1.529 variants because the B.1.1.529 lineage was recategorized as BA.1 as of February 24, 2022 (https://cov-lineages.org/lineage_list.html). For each sequence, we counted the number of undetermined nucleotides (such as N, Y, W) for whole genomes as well as *S* genes and obtained 40,739 sequences with fewer than 1,000 undetermined nucleotides in the genome and fewer than 10 undetermined nucleotides in the S-coding region. We then obtained BA.1 variant genomes that met the following criteria: 1) genomes were isolated from humans; 2) genomes did not contain any undetermined nucleotides in genomic regions corresponding to amino acid positions 371–375 in the S protein; 3) genomes were sampled from September 2021 to November 2021; and 4) genomes did not contain any of the 3 amino acid replacements in the S protein. We then selected 12 genomes and randomly selected 100 genomes that met criteria 1 and 2. We then removed Omicron genomes containing recombination sites using RDP4 v4.101^44^ because such genomes may contain artifactual reversions possibly derived from contamination of non-Omicron (mainly Delta) variants due to the low affinity of primers.^33^^,^^34^ We also checked the sequences manually, and 44 Omicron genomes were obtained.

The 44 Omicron genomes with two outgroup genomes EPI_ISL_402125 (strain Wuhan-Hu-1, B lineage) and EPI_ISL_406862 (B.1 lineage; one of the earliest sequences carrying the S D614G mutation) were aligned using FFT-NS-1 in MAFFT suite v7.407.^45^ We then deleted gapped regions in the 5' and -3′ regions. BEAST v1.10.4^46^ was used to construct a timetree under an exponential growth coalescent model using a strict molecular clock. The GTR model with the four categories of discrete gamma rate variation was used as a nucleotide substitution model.^48^^,^^49^ We ran Markov Chain Monte Carlo (MCMC) procedures with a 1 × 10^8^ chain length for all calculations, discarding the first 10% as burn-in and sampling every 10,000 replicates. The effective sample size for all run was confirmed to be larger than 200. FigTree v1.4.4 (http://tree.bio.ed.ac.uk/software/figtree/) was used to show the tree. To further determine the population history of the Omicron genomes, we generated a Bayesian skyline plot using the same model (2 × 10^8^ chain length for MCMC) and summarized the results using Tracer v1.7.1.^47^

#### Plasmid construction

Plasmids expressing the codon-optimized SARS-CoV-2 S proteins of B.1 (the parental D614G-bearing variant), Delta (B.1.617.2) and Omicron (BA.1 lineage) variants were prepared in our previous studies.^22^^,^^23^^,^^24^^,^^27^^,^^32^^,^^35^^,^^50^^,^^51^ Plasmids expressing a series of SAR-CoV-2 S mutants were generated by site-directed overlap extension PCR using the primers listed in Table S3. The resulting PCR fragment was digested with KpnI (New England Biolabs, Cat# R0142S) and NotI (New England Biolabs, Cat# R1089S) and inserted into the corresponding site of the pCAGGS vector.^38^ Nucleotide sequences were determined by DNA sequencing services (Eurofins), and the sequence data were analyzed by Sequencher v5.1 software (Gene Codes Corporation).

#### Pseudovirus assay

Lentivirus (HIV-1)-based, luciferase-expressing reporter viruses were pseudotyped with the SARS-CoV-2 spikes. HEK293T cells (500,000 cells) were cotransfected with 800 ng psPAX2-IN/HiBiT,^39^ 800 ng pWPI-Luc2,^39^ and 400 ng plasmids expressing parental S or its derivatives using TransIT-293 Transfection Reagent (Takara, Cat# MIR2700) or PEI Max (Polysciences, Cat# 24765-1) according to the manufacturer’s protocol. Two days posttransfection, the culture supernatants were harvested, and the pseudoviruses were stored at −80°C until use. The same amount of pseudoviruses [normalized to the HiBiT value measured by Nano Glo HiBiT lytic detection system (Promega, Cat# N3040)], which indicates the amount of p24 HIV-1 antigen) was inoculated into HOS-ACE2/TMPRSS2 cells and A549-ACE2 cells. At two days postinfection, the infected cells were lysed with a Bright-Glo Luciferase Assay System (Promega, cat# E2620) or a One-Glo luciferase assay system (Promega, cat# E6130) and the luminescent signal was measured using a GloMax Explorer Multimode Microplate Reader (Promega) or a CentroXS3 plate reader (Berthhold Technologies).

#### Western blot

For the blot, the HEK293 cells cotransfected with the S expression plasmids and HIV-1-based pseudovirus producing plasmids (see “pseudovirus assay” section above) or the HEK293 cells transfected with the S expression plasmids were used. To quantify the level of the cleaved S2 protein in the cells, the harvested cells were washed and lysed in lysis buffer [25 mM HEPES (pH 7.2), 10% glycerol, 125 mM NaCl, 1% Nonidet P40 substitute (Nacalai Tesque, Cat# 18558-54), protease inhibitor cocktail (Nacalai Tesque, Cat# 03969-21)]. After quantification of total protein by protein assay dye (Bio-Rad, Cat# 5000006), lysates were diluted with 2 × sample buffer [100 mM Tris-HCl (pH 6.8), 4% SDS, 12% β-mercaptoethanol, 20% glycerol, 0.05% bromophenol blue] and boiled for 10 m. Then, 10 μL samples (50 μg of total protein) were subjected to Western blot. To quantify the level of the S2 protein in the virions, 900 μL culture medium containing the pseudoviruses was layered onto 500 μL 20% sucrose in PBS and centrifuged at 20,000 g for 2 hours at 4°C. Pelleted virions were resuspended in 1× NuPAGE LDS sample buffer (Thermo Fisher Scientific, Cat# NP0007) containing 2% β-mercaptoethanol and incubated at 70°C for 10 m. For protein detection, the following antibodies were used: mouse anti-SARS-CoV-2 S monoclonal antibody (clone 1A9, GeneTex, Cat# GTX632604, 1:10,000), mouse anti-HIV-1 p24 monoclonal antibody (183-H12-5C, obtained from the HIV Reagent Program, NIH, Cat# ARP-3537, 1:2,000), rabbit anti-beta actin (ACTB) monoclonal antibody (clone 13E5, Cell Signalling, Cat# 4970, 1:5,000), mouse anti-tubulin (TUBA) monoclonal antibody (clone DM1A, Sigma-Aldrich, Cat# T9026, 1:10,000), horseradish peroxidase (HRP)-conjugated horse anti-mouse IgG antibody (Cell Signaling, Cat# 7076S, 1:2,000), HRP-conjugated donkey anti-rabbit IgG polyclonal antibody (Jackson ImmunoResearch, Cat# 711-035-152, 1:10,000) and HRP-conjugated donkey anti-mouse IgG polyclonal antibody (Jackson ImmunoResearch, Cat# 715-035-150, 1:10,000). Chemiluminescence was detected using SuperSignal West Femto Maximum Sensitivity Substrate (Thermo Fisher Scientific, Cat# 34095), SuperSignal West Atto Ultimate Sensitivity Substrate (Thermo Fisher Scientific, Cat# A38554) or Western BLoT UltraSensitive HRP Substrate (Takara, Cat# T7104A) according to the manufacturer’s instruction. Bands were visualized using an Amersham Imager 600 (GE Healthcare) or iBright FL1500 Imaging System (Thermo Fisher Scientific), and the band intensity was quantified using Image Studio Lite v5.2 (LI-COR Biosciences) or Fiji software v2.2.0 (ImageJ). Uncropped blots are shown in Figure S4.

#### SARS-CoV-2 S-based fusion assay

The SARS-CoV-2 S-based fusion assay^24^^,^^52^ utilizes a dual split protein (DSP) encoding *Renilla* luciferase and *GFP* genes; the respective split proteins, DSP_8-11_ and DSP_1-7_, are expressed in effector and target cells by transfection. Briefly, on day 1, effector cells (i.e., S-expressing cells) and target cells (see below) were prepared at a density of 0.6–0.8 × 10^6^ cells in a 6-well plate. To prepare effector cells, HEK293 cells were cotransfected with the S expression plasmids (400 ng) and pDSP_8-11_ (400 ng) using TransIT-LT1 (Takara, Cat# MIR2300). To prepare target cells, HEK293 cells were cotransfected with pC-ACE2 (200 ng) and pDSP_1-7_ (400 ng). Target HEK293 cells in selected wells were cotransfected with pC-TMPRSS2 (40 ng) in addition to the plasmids above. HEK293-ACE2 cells and HEK293-ACE2/TMPRSS2 cells were transfected with pDSP_1-7_ (400 ng). On day 3 (24 h posttransfection), 16,000 effector cells were detached and reseeded into 96-well black plates (PerkinElmer, Cat# 6005225), and target HEK293 cells were reseeded at a density of 1,000,000 cells/2 mL/well in 6-well plates. On day 4 (48 h posttransfection), target cells were incubated with EnduRen live cell substrate (Promega, Cat# E6481) at 37°C for 3 h and then detached, and 32,000 target cells were added to a 96-well plate with effector cells. *Renilla* luciferase activity was measured at the indicated time points using Centro XS3 LB960 (Berthhold Technologies). To measure the surface expression level of S protein, effector cells were stained with rabbit anti-SARS-CoV-2 S S1/S2 polyclonal antibody (Thermo Fisher Scientific, Cat# PA5-112048, 1:100). Normal rabbit IgG (SouthernBiotech, Cat# 0111-01, 1:100) was used as negative controls, and APC-conjugated goat anti-rabbit IgG polyclonal antibody (Jackson ImmunoResearch, Cat# 111-136-144, 1:50) was used as a secondary antibody. Surface expression level of S protein was measured using FACS Canto II (BD Biosciences) and the data were analyzed using FlowJo software v10.7.1 (BD Biosciences). To calculate fusion activity, *Renilla* luciferase activity was normalized to the MFI of surface S proteins. The normalized value (i.e., *Renilla* luciferase activity per the surface S MFI) is shown as fusion activity.

#### SARS-CoV-2 reverse genetics

To generate recombinant SARS-CoV-2 by circular polymerase extension reaction (CPER),^25^^,^^24^ 9 DNA fragments encoding the partial genome of SARS-CoV-2 (strain WK-521, PANGO lineage A; GISAID ID: EPI_ISL_408667)^37^ were prepared by PCR using PrimeSTAR GXL DNA polymerase (Takara, Cat# R050A). A linker fragment encoding hepatitis delta virus ribozyme, bovine growth hormone poly A signal and cytomegalovirus promoter was also prepared by PCR. The corresponding SARS-CoV-2 genomic region and the PCR templates and primers used for this procedure are summarized in Table S3. The 10 obtained DNA fragments were mixed and used for CPER.^25^ To prepare GFP-expressing replication-competent recombinant SARS-CoV-2, we used fragment 9, in which the *GFP* gene was inserted in the *ORF7a* frame, instead of the authentic F9 fragment (Table S3).^25^

To generate chimeric recombinant SARS-CoV-2 (Figures 1G and 5A), mutations were inserted in fragment 8 by site-directed overlap extension PCR or the GENEART site-directed mutagenesis system (Thermo Fisher Scientific, Cat# A13312) according to the manufacturer’s protocol with the primers listed in Table S3. Recombinant SARS-CoV-2 that bears B.1 S [rB.1 S-GFP (virus I)] or Omicron S [rOmicron S-GFP (virus II)] was prepared in our previous studies.^23^^,^^32^ Nucleotide sequences were determined by a DNA sequencing service (Fasmac), and the sequence data were analyzed by Sequencher v5.1 software (Gene Codes Corporation).

To produce chimeric recombinant SARS-CoV-2, the CPER products were transfected into HEK293-C34 cells using TransIT-LT1 (Takara, Cat# MIR2300) according to the manufacturer’s protocol. At 1 d posttransfection, the culture medium was replaced with Dulbecco’s modified Eagle’s medium (high glucose) containing 2% FCS, 1% PS and doxycycline (1 μg/mL; Takara, Cat# 1311N). At 7 d posttransfection, the culture medium was harvested and centrifuged, and the supernatants were collected as the seed virus. To remove the CPER products (i.e., SARS-CoV-2-related DNA), 1 mL of the seed virus was treated with 2 μL TURBO DNase (Thermo Fisher Scientific, Cat# AM2238) and incubated at 37°C for 1 h. Complete removal of the CPER products (i.e., SARS-CoV-2-related DNA) from the seed virus was verified by PCR. The working virus stock was prepared from the seed virus as described below (see “SARS-CoV-2 preparation and titration” section).

#### SARS-CoV-2 preparation and titration

To prepare the working virus stocks of chimeric recombinant SARS-CoV-2,^25^^,^^24^ 20 μL of the seed virus was inoculated into VeroE6/TMPRSS2 cells (5,000,000 cells in a T-75 flask). One hour post infection (h.p.i.), the culture medium was replaced with DMEM (low glucose) (Wako, Cat# 041-29775) containing 2% FBS and 1% PS. At 3 d.p.i., the culture medium was harvested and centrifuged, and the supernatants were collected as the working virus stock.

The titer of the prepared working virus was measured as the 50% tissue culture infectious dose (TCID_50_). Briefly, one day before infection, VeroE6/TMPRSS2 cells (10,000 cells) were seeded into a 96-well plate. Serially diluted virus stocks were inoculated into the cells and incubated at 37°C for 4 days. The cells were observed under microscopy to judge the CPE appearance. The value of TCID_50_/mL was calculated with the Reed–Muench method.^53^

To verify the sequence of chimeric recombinant SARS-CoV-2, viral RNA was extracted from the working viruses using a QIAamp viral RNA mini kit (Qiagen, Cat# 52906) and viral genome sequence was analyzed as described above (see "viral genome sequencing" section above). In brief, the viral sequences of *GFP*-encoding recombinant SARS-CoV-2 (strain WK-521; GISIAD ID: EPI_ISL_408667)^25^^,^^37^ that harbor the *S* genes of respective variants were used for the reference. Information on the unexpected mutations detected is summarized in Table S4, and the raw data are deposited in DDBJ Sequence Read Archive (accession number: PRJDB13805).

#### SARS-CoV-2 infection

One day before infection, VeroE6/TMPRSS2 cells (10,000 cells) were seeded into a 96-well plate. SARS-CoV-2 (100 TCID_50_, m.o.i. 0.01) was inoculated and incubated at 37°C for 1 hour. The infected cells were washed, and 180 μL of culture medium was added. The culture supernatant (10 μL) was harvested at the indicated timepoints and used for RT–qPCR to quantify the viral RNA copy number (see “RT–qPCR” section below).

#### RT–qPCR

Five microliters of culture supernatant was mixed with 5 μL of 2 × RNA lysis buffer [2% Triton X-100 (Nacalai Tesque, Cat# 35501-15), 50 mM KCl, 100 mM Tris-HCl (pH 7.4), 40% glycerol, 0.8 U/μL recombinant RNase inhibitor (Takara, Cat# 2313B)] and incubated at room temperature for 10 minutes. RNase-free water (90 μL) was added, and the diluted sample (2.5 μL) was used as the template for real-time RT-PCR performed according to the manufacturer’s protocol using the OneStep TB Green PrimeScript PLUS RT-PCR kit (Takara, Cat# RR096A) and the following primers: Forward *N*, 5′-AGC CTC TTC TCG TTC CTC ATC AC-3′; and Reverse *N*, 5′-CCG CCA TTG CCA GCC ATT C-3′. The viral RNA copy number was standardized with a SARS-CoV-2 direct detection RT-qPCR kit (Takara, Cat# RC300A). Fluorescent signals were acquired using QuantStudio 3 Real-Time PCR system (Thermo Fisher Scientific), Thermal Cycler Dice Real Time System III (Takara), CFX Connect Real-Time PCR Detection system (Bio-Rad), Eco Real-Time PCR System (Illumina), qTOWER3 G Real-Time System (Analytik Jena) or 7500 Real-Time PCR System (Thermo Fisher Scientific).

#### Fluorescence microscopy

One day before infection, VeroE6/TMPRSS2 cells (10,000 cells) were seeded into 96-well, glass bottom, black plates and infected with SARS-CoV-2 (100 TCID_50_, m.o.i. 0.01). At 24, 48, and 72 h.p.i., GFP fluorescence was observed under an All-in-One Fluorescence Microscope BZ-X800 (Keyence) in living cells, and a 13-square-millimeter area of each sample was scanned. under the same parameters. Images were reconstructed using an BZ-X800 analyzer software (Keyence), and the area and the fluorescent intensity of the GFP-positive cells was measured using this software.

#### Plaque assay

One day before infection, VeroE6/TMPRSS2 cells (100,000 cells) were seeded into a 24-well plate and infected with SARS-CoV-2 (1, 10, 100 and 1,000 TCID_50_) at 37°C for 2 hours. Mounting solution containing 3% FBS and 1.5% carboxymethyl cellulose (Wako, Cat# 039-01335) was overlaid, followed by incubation at 37°C. At 3 d.p.i., the culture medium was removed, and the cells were washed with PBS three times and fixed with 4% paraformaldehyde phosphate buffer solution (Nacalai Tesque, Cat# 09154-85). The fixed cells were washed with tap water, dried, and stained with staining solution [0.1% methylene blue (Nacalai Tesque, Cat# 22412-14) in water] for 30 minutes. The stained cells were washed with tap water and dried, and the size of plaques was measured using Adobe Photoshop 2021 v22.4.1 (Adobe).

#### Neutralization assay

For neutralization assay,^35^ pseudoviruses were prepared as described above (see “pseudovirus assay” section). For the neutralization assay, the SARS-CoV-2 S pseudoviruses (counting ∼20,000 relative light units) were incubated with serially diluted (40-fold or 120-fold to 29,160-fold dilution at the final concentration) heat-inactivated sera at 37°C for 1 hour. Pseudoviruses without sera were included as controls. Then, an 80 μL mixture of pseudovirus and serum/antibody was added to HOS-ACE2/TMPRSS2 cells (10,000 cells/50 μL) in a 96-well white plate. At 2 d.p.i., pseudovirus infectivity was measured as described above (see “pseudovirus assay” section). The assay of each serum was performed in triplicate, and the 50% neutralization titer (NT_50_) was calculated using Prism 9 (GraphPad Software).

#### Protein structure

All protein structural analyses were performed using the PyMOL molecular graphics system v2.5.0 (Schrödinger). The cryo-EM structures of SARS-CoV-2 D614G (B.1 lineage) S (PDB: 7KRQ)^28^ and Omicron S (PDB: 7T9J)^29^ were used. To predict inter-subunit interaction of the Omicron S trimer, each subunit of the D614G S trimer was replaced with the Omicron S monomer.^29^ The distance between F375 and H505 was measured using the PyMOL molecular graphics system v2.5.0 (Schrödinger).

#### Yeast surface display

For yeast surface display,^26^ the carboxypeptidase domain of human ACE2 (residues 18–740) was expressed in Expi293F cells and purified by a 5-mL HisTrap Fast Flow column (Cytiva, Cat# 17-5255-01) and Superdex 200 16/600 (Cytiva, Cat# 28-9893-35) using an ÄKTA pure chromatography system (Cytiva), and the purified soluble ACE2 was labelled with CF640R (Biotium, Cat# 92108). Protein quality was verified using a Tycho NT.6 system (NanoTemper) and ACE2 activity assay kit (SensoLyte, Cat# AS-72086).

An enhanced yeast display platform for SARS-CoV-2 S RBD [wild-type (B.1.1), residues 336–528] yeast surface expression was established using *Saccharomyces cerevisiae* EBY100 strain and pJYDC1 plasmid (Addgene, Cat# 162458) as previously described.^14^^,^^23^^,^^24^^,^^26^^,^^27^ To prepare a series of SARS-CoV-2 S RBD mutants, the site-directed mutagenesis was performed using the KAPA HiFi HotStart ReadyMix kit (Roche, Cat# KK2601) by restriction enzyme-free cloning procedure.^54^ Primers for mutagenesis are listed in Table S3.

The binding affinities of SARS-CoV-2 S RBDs to human ACE2 were determined by flow cytometry titration experiments. The CF640R-labelled ACE2 at 12–14 different concentrations (200 nM to 13 pM in PBS supplemented with bovine serum albumin at 1 mg/mL) per measurement were incubated with expressed yeast aliquots and 10 nM bilirubin (Sigma-Aldrich, Cat# 14370-1G) and analyzed by using FACS S3e Cell Sorter device (Bio-Rad). The background binding subtracted fluorescent signal was fitted to a standard noncooperative Hill equation by nonlinear least-squares regression using Python v3.7 (https://www.python.org) as previously described.^26^

### Quantification and statistical analysis

In the single timepoint experiments, statistical significance was tested using a two-sided Student’s *t* test (Figures 1B, 1E, 3B, 4B, 4E, 6D, 6E and S1A), a two-sided paired *t* test (Figures 1D, 4D, 6B and 6C), a two-sided Mann–Whitney *U* test (Figures 1J, 1K, 5D, 5E), or a two-sided Wilcoxon signed-rank test (Figure 2). The tests above were performed using Prism 9 software v9.1.1 (GraphPad Software).

In the time-course experiments (Figures 1F, 1H, 1I, 4F, 5B, 5C, 6F, and 6G), a multiple regression analysis including experimental conditions (i.e., the types of infected viruses) as explanatory variables and timepoints as qualitative control variables was performed to evaluate the difference between experimental conditions thorough all timepoints. *p* value was calculated by a two-sided Wald test. Subsequently, familywise error rates (FWERs) were calculated by the Holm method. These analyses were performed in R v4.1.2 (https://www.r-project.org/).

In Figures 1J, 1I, 5C, 5D and S1B, assays were performed in triplicate. Photographs shown are the representatives of >18 fields of view taken for each sample.

## Data Availability

RNA-seq data generated in this study were deposited on the DDBJ Sequence Read Archive (https://www.ddbj.nig.ac.jp/dra/) with the accession numbers DRR385305 - DRR385309 with BioProject ID PRJDB13805. GISAID IDs used in this study was available at the following https://doi.org/10.55876/gis8.221004su. Any additional information required to reanalyze the data reported in this paper is available from the lead contact upon request.

## Supplemental information

Document S1. Figures S1–S4

Table S1. Human sera used in this study, related to Figure 2

Table S2. Mutations detected in the Omicron RBD, related to Figure 3

Table S3. Primers used in this study, related to Figures 1 and 3–6

Table S4. Summary of the mutations detected in the working virus stocks compared to the original sequences, related to Figures 1 and 5
