# Supplementary material for: The SARS-CoV-2 spike S375F mutation characterizes the Omicron BA.1 variant
Source: iScience. 2022 Dec 5;25(12):105720. doi: 10.1016/j.isci.2022.105720 (PMC9719929; doi:10.1016/j.isci.2022.105720)
Supplement: Document S1. Figures S1–S4 [file mmc1.pdf]

iScience, Volume *25*

Supplemental information

The SARS-CoV-2 spike S375F mutation characterizes the Omicron BA.1 variant

Izumi Kimura, Daichi Yamasoba, Hesham Nasser, Jiri Zahradnik, Yusuke Kosugi, Jiaqi Wu, Kayoko Nagata, Keiya Uriu, Yuri L. Tanaka, Jumpei Ito, Ryo Shimizu, Toong Seng Tan, Erika P. Butlertanaka, Hiroyuki Asakura, Kenji Sadamasu, Kazuhisa Yoshimura, Takamasa Ueno, Akifumi Takaori-Kondo, Gideon Schreiber, the Genotype to Phenotype Japan (G2P-Japan) Consortium, Mako Toyoda, Kotaro Shirakawa, Takashi Irie, Akatsuki Saito, So Nakagawa, Terumasa Ikeda, and Kei Sato

Supplementary Information

The SARS-CoV-2 spike S375F mutation characterizes the Omicron BA.1 variant

Izumi Kimura, Daichi Yamasoba, Hesham Nasser, Jiri Zahradnik, Yusuke Kosugi, Jiaqi Wu, Kayoko Nagata, Keiya Uriu, Yuri L Tanaka, Jumpei Ito, Ryo Shimizu, Toong Seng Tan, Erika P Butlertanaka, Hiroyuki Asakura, Kenji Sadamasu, Kazuhisa Yoshimura, Takamasa Ueno, Akifumi Takaori-Kondo, Gideon Schreiber, The Genotype to Phenotype Japan (G2P-Japan) Consortium, Mako Toyoda, Kotaro Shirakawa, Takashi Irie, Akatsuki Saito, So Nakagawa, Terumasa Ikeda, Kei Sato

Supplementary Figures S1-S4
Supplementary Tables S1-S4

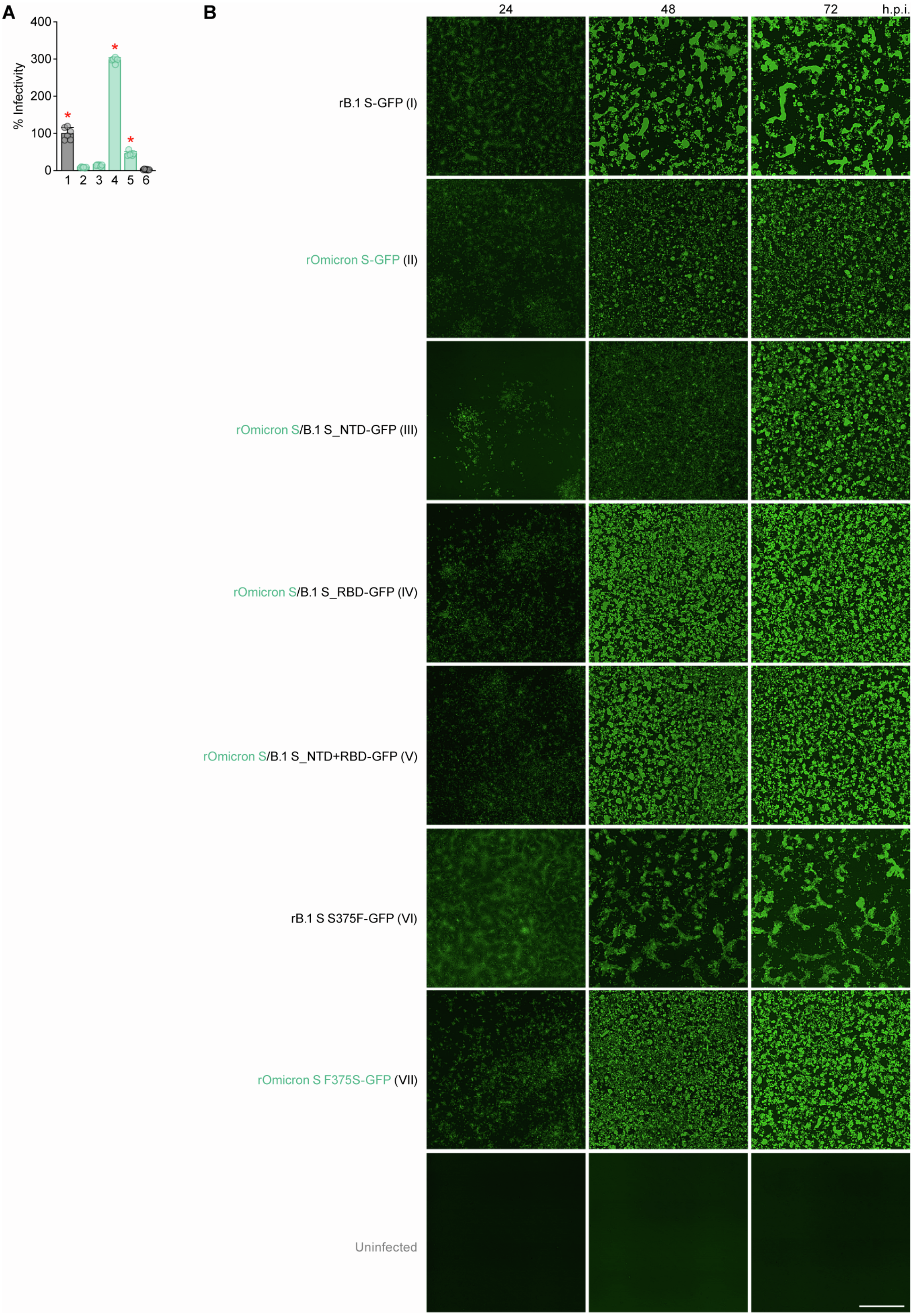

Figure S1. Virological properties conferred by the Omicron RBD, related to Figures 1 and 5.

(**A**) Pseudovirus assay. HIV-1-based reporter viruses pseudotyped with SARS-CoV-2 S chimeras (summarized in **Figure 1A**) were prepared. The pseudoviruses were inoculated into

A549-ACE2 cells at 100 ng HIV-1 p24 antigen, and the percentages of infectivity compared to that of the virus pseudotyped with B.1 S (spike 1) are shown.
(**B**) Fluorescence microscopy to measure the GFP area were measured in infected VeroE6/TMPRSS2 cells (m.o.i. 0.01) at 24, 48, and 72 h.p.i. The panels at 48 h.p.i. are identical to those shown in **Figures 1J and 5D**. Scale bar, 500 μm.
In **A**, assays were performed in sextuplicate, and data are expressed as the mean with SD. Each dot indicates the result of an individual replicate. Statistically significant differences (*P<0.05) versus Omicron S (pseudovirus 2) were determined by two-sided Student's t test.

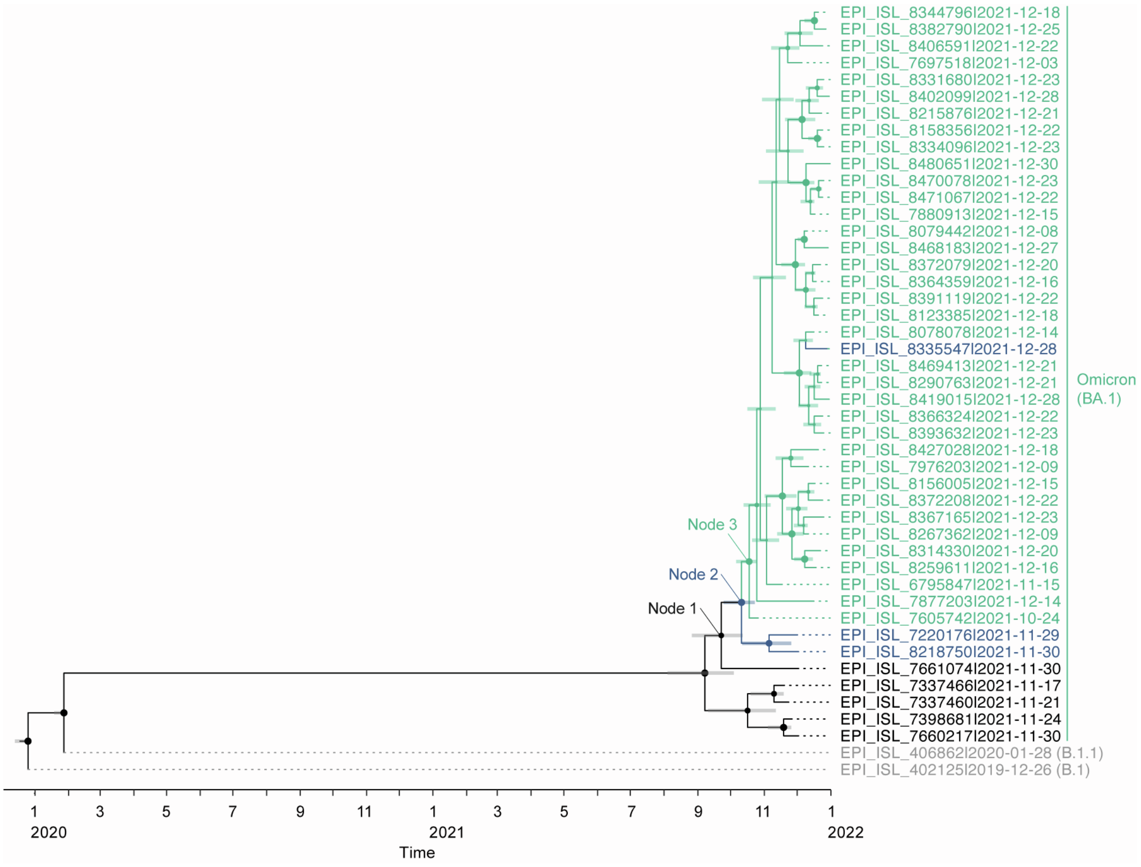

Figure S2. Molecular phylogenetic of Omicron, related to Figure 3.

A timetree of 44 Omicron variants and two outgroups (B and B.1 lineages) with GISAID ID, PANGO lineage and sampling date. The topology of the phylogenetic tree is identical to that shown in **Figure 3C**, top. Green, Omicron variants containing S371L, S373P and S375F mutations; blue, Omicron variants containing the S371L and S373P mutations; black, Omicron variants without the S371L/S373P/S375F mutations; and gray, the two outgroups (B and B.1 lineages). Bars on each internal node indicate the 95% HPD interval of estimation time. The size of the circle on each internal node is proportional to the value of posterior probability.

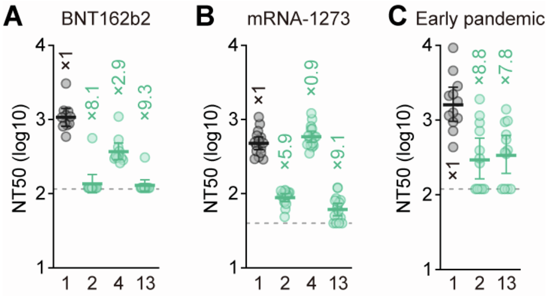

Figure S3. Immune resistance of the Omicron S S371L/S373P/S375F mutant, related to Figure 4.

Neutralization assays were performed with pseudoviruses harboring a series of S proteins (summarized in **Figures 1A and 4A**). The numbers are identical to those in **Figures 1A and 4A**. Vaccinated sera [BNT162b2 (**A**, 11 donors); or mRNA-1273 (**B**, 16 donors)] and convalescent sera of individuals infected with an early pandemic virus (until May 2020) (**C**, 12 donors) were used. The list of sera used in this experiment is shown in **Table S1**. Each serum sample was tested in triplicate to determine the 50% neutralization titer (NT50). Each dot represents one NT50 value, and the geometric mean and 95% CI are shown. The numbers indicate the fold changes of resistance versus each antigenic variant. Horizontal gray lines indicate the detection limit of each assay (120 for **A and C**; 40 for **B**).

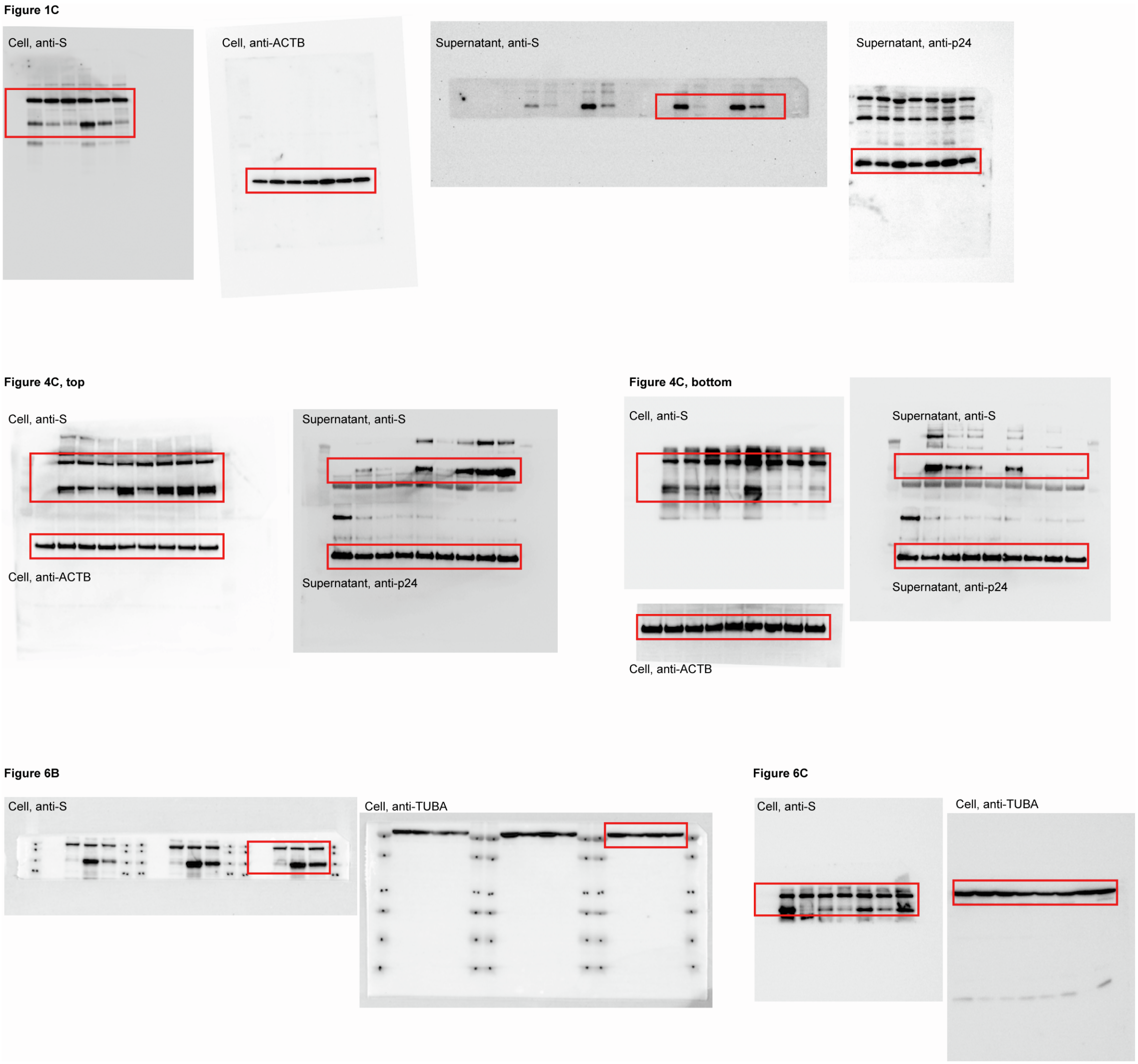

Figure S4. Uncropped images of western blots, related to Figures 1, 4 and 6. Uncropped images of representative western blots. The areas marked in red are cropped and shown in the indicated figures.
